# An Injectable ROS‐Responsive Nanozyme Hydrogel Regulates the Uterine Microenvironment to Prevent Intrauterine Adhesions

**DOI:** 10.1002/advs.77510

**Published:** 2026-08-30

**Authors:** Peixian Cheng, Jian Song, Yuxin Li, Ming Chen, Zeshen Liang, Binyao Qiu, Xuemin Liu, Xuetao Shi, Yong Fan

**Affiliations:** ^1^ Department of Obstetrics and Gynecology, Guangdong Provincial Key Laboratory of Major Obstetric Diseases, Guangdong Provincial Clinical Research Center for Obstetrics and Gynecology, Guangdong‐Hong Kong‐Macao Greater Bay Area Higher Education Joint Laboratory of Maternal‐Fetal Medicine The Third Affiliated Hospital, Guangzhou Medical University Guangzhou China; ^2^ Research Centre of Basic Integrative Medicine, School of Basic Medical Sciences Guangzhou University of Chinese Medicine Guangzhou China; ^3^ Department of Obstetrics and Gynecology The First Affiliated Hospital of Naval Medical University, Changhai Hospital Shanghai China; ^4^ National Engineering Research Centre for Tissue Restoration and Reconstruction South China University of Technology Guangzhou China; ^5^ Gynecology Department The Second Affiliated Hospital, Guangzhou Medical University Guangzhou China; ^6^ Key Laboratory of New Drug Design, School of Pharmacy East China University of Science and Technology Shanghai China

**Keywords:** endometrial regeneration, intrauterine adhesions, metal‐phenolic nanozyme, ROS‐responsive hydrogel, uterine microenvironment remodeling

## Abstract

Intrauterine injury triggers a self‐reinforcing cycle of inflammation, oxidative stress, and fibrosis that culminates in intrauterine adhesions (IUA), severely impairing women's reproductive health. Current treatments, including hysteroscopic adhesiolysis combined with hormonal therapy or physical barriers, show high recurrence and cannot simultaneously target these interconnected pathways. Here, we developed an injectable ROS‐responsive hydrogel (GPP) loaded with cerium–tannic acid metal‐phenolic nanozymes (CeTA) that exhibit superoxide dismutase (SOD)‐ and catalase (CAT)‐mimetic activities. Unlike passive barriers, its boronate ester‐crosslinked network remains stable under physiological conditions but is selectively cleaved in ROS‐rich inflammatory microenvironments, enabling on‐demand, lesion‐localized CeTA delivery. In vitro, by scavenging excess superoxide and hydrogen peroxide, CeTA alleviated oxidative stress, restored mitochondrial membrane potential, promoted macrophage repolarization from the pro‐inflammatory M1 to the anti‐inflammatory M2 phenotype, and reversed TGF‐β1‐induced fibrosis in endometrial stromal cells. In a rat IUA model, CeTA@GPP hydrogel restored uterine antioxidant enzyme activity, suppressed TGF‐β1 expression and myofibroblast activation, and enhanced cell proliferation and angiogenesis; transcriptomic profiling confirmed coordinated down‐regulation of the inflammation, oxidative stress, and fibrosis pathways. Accordingly, CeTA@GPP hydrogel promoted structural and functional endometrial regeneration and improved endometrial receptivity. By integrating minimally invasive injectability, physical barrier protection, and intelligent ROS‐responsive multi‐target bioactivity, CeTA@GPP hydrogel provides a promising strategy for IUA.

## Graphical abstract

The injectable CeTA@GPP hydrogel is administered via intrauterine injection, forming a protective barrier. In the high ROS inflammatory microenvironment of injured endometrium, boronate ester cleavage triggers responsive CeTA release and local enrichment. The hydrogel targets inflammation, oxidative stress, and fibrosis via spatial barrier protection and multi‐target regulation, achieving precise IUA intervention.

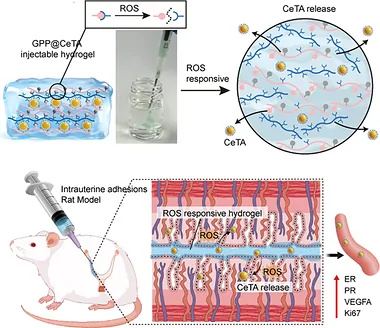

## 1 Introduction

Unlike most mucosal tissues, which heal by physiological scar‐free regeneration, the injured endometrium frequently defaults to pathological fibrotic repair following mechanical or infectious injury [1, 2, 3]. This maladaptive repair gives rise to intrauterine adhesions (IUA), characterized by fibrous adhesive bands between the anterior and posterior uterine walls [4] that partially or completely obliterate the cavity, leading to menstrual disorders, recurrent miscarriage, and infertility [5, 6]. The incidence of IUA ranges from 16% to 24% after curettage and rises to 31% to 45% following hysteroscopic myomectomy [7]. Critically, ongoing pregnancy (58%) and live birth (54%) rates in IUA patients are markedly lower than in unaffected women (90% and 84%, respectively), severely impairing reproductive outcomes [8].

The core pathological feature of IUA is a self‐reinforcing cascade of inflammation, oxidative stress, and fibrosis triggered by basal layer injury, with macrophage polarization and ROS overproduction as its two pivotal nodes. Persistent inflammation, driven mainly by an imbalanced shift toward the M1 macrophage phenotype, precedes and positively correlates with fibrosis severity [9], M1 macrophages secrete pro‐inflammatory cytokines (TNF‐α, IL‐1β) and pro‐fibrotic mediators (CCL5, SPP1) that promote fibroblast‐to‐myofibroblast transformation and excessive collagen deposition [10, 11]. By contrast, under normal conditions, macrophages secrete cytokines and bioactive factors that regulate endometrial differentiation, vascularization, and glandular development, maintaining receptivity and immune homeostasis [12], while uterine CD4^+^ and CD8^+^ T cells also contribute to local immune regulation [13]. Concurrently, massive ROS accumulation impairs cell membranes, mitochondria, and DNA [14] and suppresses stromal cell proliferation and angiogenesis [15]; it further activates pro‐fibrotic signaling pathways (e.g., TGF‐β, MAPK, Notch) and induces epithelial‐mesenchymal transition in endometrial epithelial cells [16]. Together, these intertwined processes lock IUA into a vicious cycle of progression.

Although hysteroscopic adhesiolysis combined with estrogen therapy and physical barriers is the standard treatment for IUA [17], postoperative re‐adhesion remains high (40%–62.5%) [3, 18]. Moreover, long‐term systemic hormone use raises thrombotic and metabolic risks, and physical barriers such as uterine balloons struggle to adapt to the irregular cavity and may trigger mechanical inflammation [19, 20]. Barrier biomaterials, including hydrogel membranes, have likewise been explored but entail practical drawbacks such as difficult handling and the need for secondary removal surgery [21]. Injectable hydrogels circumvent these issues: their liquid precursors can be delivered by minimally invasive injection and form three‐dimensional networks that conform to irregular uterine contours, serving as both physical barriers and drug carriers [19, 22]. Clinically applied hyaluronic acid hydrogels, however, fail to provide sustained protection owing to rapid degradation [23, 24]. Although crosslinking strategies improve resistance to enzymatic and oxidative degradation [25], such passive matrices still lack active responsiveness to the pathological microenvironment [26]. An ideal smart hydrogel should not only fill the defect or deliver drugs but also respond to local signals to remodel the microenvironment [27, 28, 29]. Responsive platforms triggered by photothermal or pH cues, including MXene based materials [30] and adaptive dual crosslinked systems that modulate disease related signaling [31], have made notable progress in tissue repair. However, photothermal and pH triggers are poorly suited to the enclosed, optically inaccessible uterine cavity, whereas oxidative stress is a central driver of IUA progression that also directly impairs oocyte quality [32], providing an intrinsic, disease‐specific trigger and making restoration of redox homeostasis a compelling therapeutic strategy. Existing redox tools, such as the SOD2‐Res@CVs engineered vesicle system, achieve multi‐mechanism antioxidant intervention but suffer from complex preparation, limited in vivo stability, and lower SOD/CAT efficiency than the Ce^3+^/Ce^4+^ cycle [33]. Leveraging a ROS‐responsive hydrogel delivering nucleus pulposus cell membrane‐coated black phosphorus@cerium oxide nanozymes allows for concurrent long‐term ROS elimination and blockade of the IL‐6/STAT3 axis [34]. Other studies have adopted photo‐responsive hydrogels incorporated with dendrimer (G3)‐functionalized nanoceria (G3@nCe) to achieve outstanding bone regeneration efficacy [35]. Driven by H_2_O_2_, nanoceria continuously scavenges excess ROS through this reversible Ce^3+^/Ce^4+^ cycle; we therefore selected cerium‐based nanozymes as the core functional component for their SOD‐ and CAT‐like and anti‐inflammatory activities [36]. Antioxidant, anti‐inflammatory, and anti‐fibrotic hydrogels (e.g., PXNT), oxidative‐stress‐regulating nanoparticles (LPA) and synergistic functional material systems integrating drug delivery as well as dual‐target scavenging of ROS and cfDNA further support this rationale [37, 38, 39]. Although substantial progress has been made in the development of injectable hydrogels, achieving intelligent responsiveness together with coordinated modulation of inflammation, oxidative stress, and fibrosis remains challenging, underscoring the urgent need for multifunctional, microenvironment‐responsive strategies.

To overcome the inherent limitations of conventional hydrogels, namely inadequate stimulus‐responsiveness and restricted therapeutic targets, this study developed a ROS‐responsive smart hydrogel (CeTA@GPP) based on dynamic boronate ester crosslinking and loaded with cerium‐tannic acid nanozymes (CeTA) [40, 41], designed to intervene precisely in the pathological process of IUA through the dual mechanism of space occupying protection and multi‐target regulation. Phenylboronic acid modified gelatin (Gel‐PBA) is dynamically crosslinked with polyvinyl alcohol (PVA) and CeTA via boronic ester bonds [42] to form a three‐dimensional network in which these bonds act as ROS‐sensitive switches [43]. Among endogenous ROS, the highly stable H_2_O_2_ attacks the electron‐deficient boron center at physiological pH to form a tetrahedral boronate that subsequently hydrolyzes to release the payload [44]; accordingly, the bonds remain intact under physiological conditions but cleave selectively in ROS‐rich inflammatory milieus, triggering on‐demand release and localized enrichment of CeTA at lesion sites. In vitro, the multifunctional CeTA nanozymes scavenged ROS via SOD‐ and CAT‐like activities, restored mitochondrial membrane potential, promoted M1 to M2 macrophage polarization, and inhibited fibrosis in endometrial stromal cells, collectively interrupting the inflammation, oxidative stress and fibrosis cascade. In a rat IUA model, CeTA@GPP hydrogel significantly alleviated inflammation and reduced fibrosis, effectively preventing adhesion formation. In summary, this work presents an integrated strategy combining injectable delivery, smart responsiveness, and multi‐target intervention, offering a promising biomaterial solution for endometrial repair.

## 2 Results and Discussion

### 2.1 Synthesis and Characterization of CeTA

**FIGURE 1:**
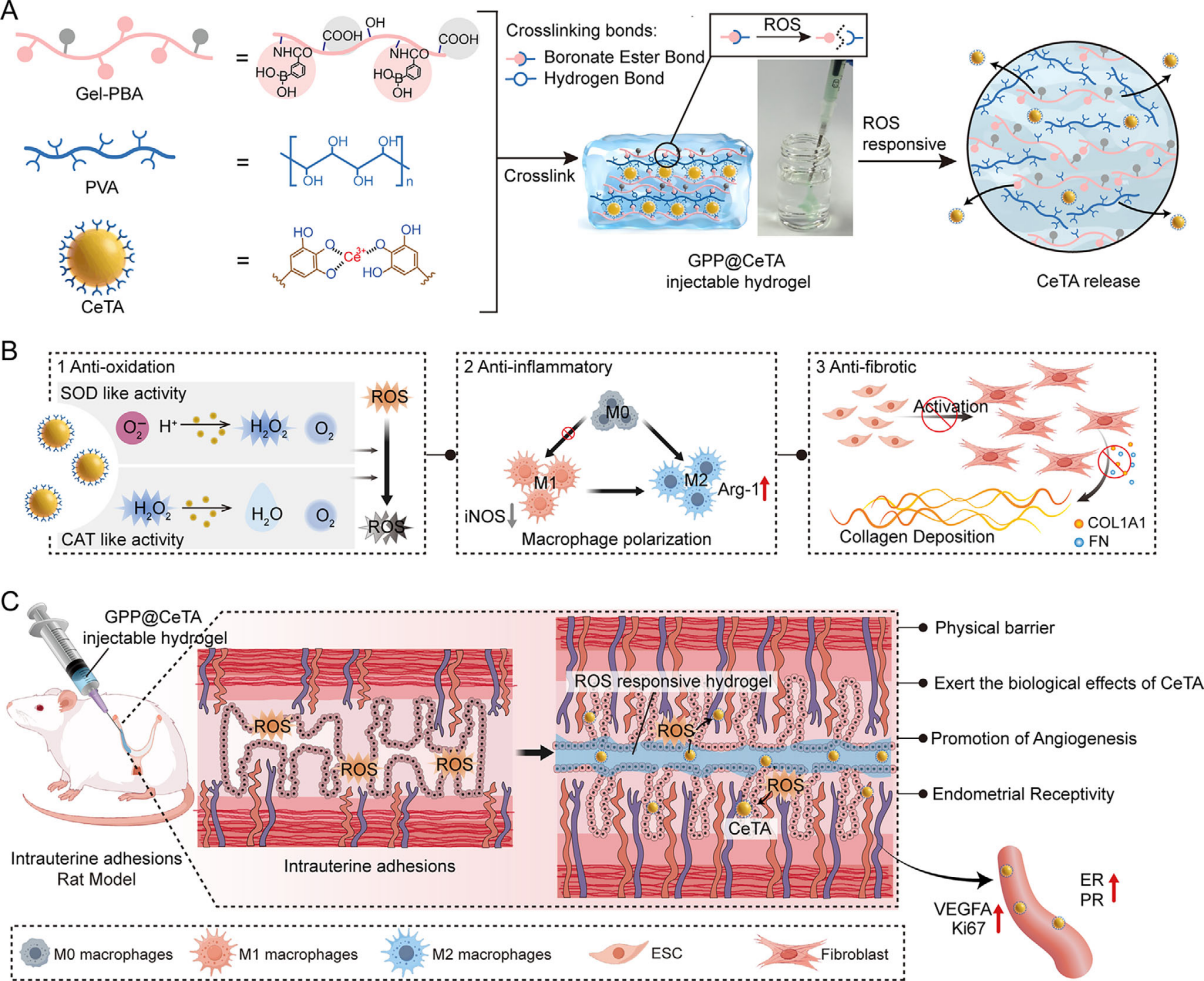
Schematic illustration of the preparation of injectable CeTA@GPP hydrogel and its application for IUA prevention.

The core pathological mechanism of IUA is the inflammation, oxidative stress and fibrosis cascade [45]. To precisely intervene in this pathological process, we designed and synthesized CeTA, which possesses multiple antioxidant enzyme activities to efficiently scavenge ROS [40, 46, 47] (Figure 1). TA, a natural polyphenolic compound, not only exhibits anti‐inflammatory, antioxidant, and anti‐fibrotic activities, but also forms stable coordination bonds with metal ions through its abundant phenolic hydroxyl groups [48, 49, 50]. Studies have demonstrated that TA modulates macrophage polarization to alleviate inflammation and inhibits TGF‐β1‐induced fibroblast activation by modulating Smad and Erk signaling pathways [51, 52]. Concurrently, cerium‐based nanomaterial, owing to the reversible redox transition between Ce^3^^+^ and Ce^4^^+^, can mimic the enzymatic activities of SOD and CAT, effectively neutralizing ROS and shielding cells from oxidative damage [36, 53].

**FIGURE 2:**
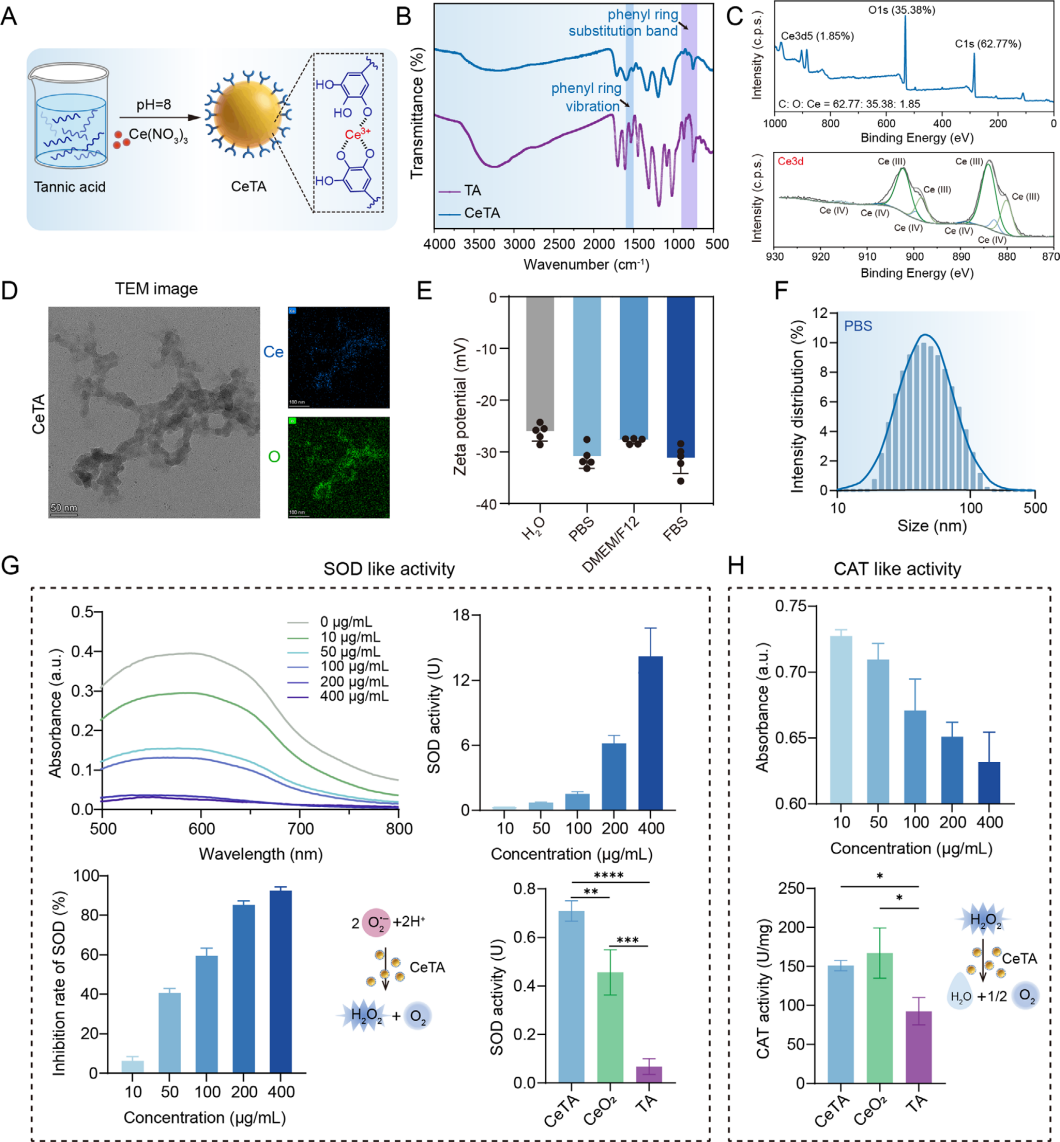
Synthesis and characterization of CeTA. (A) Schematic synthesis of CeTA. (B) FTIR patterns of TA and CeTA. (C) XPS pattern of CeTA and Ce3d of CeTA. (D) FE‐TEM image (Scale bar = 50 nm) and EDS elemental mapping images (Scale bar = 100 nm) of CeTA. (E) Zeta potential of CeTA in different solvents. (F) The hydrodynamic diameter distribution of CeTA in PBS. (G) SOD‐like activity of different concentrations of CeTA and comparison of SOD‐like activity of CeTA, CeO2, and TA at 50 µg/mL (n = 3). (H) CAT‐like activity of different concentrations of CeTA and comparison of CAT‐like activity of CeTA, CeO2, and TA at 50 µg/mL (n = 3). All data are expressed as mean ± SD. (*p < 0.05, **p < 0.01, ***p < 0.001, ****p < 0.0001).

This study successfully synthesized CeTA via a self‐assembly approach (Figure 2A). We first utilized FTIR to validate the coordination interaction between TA and Ce ions. The FTIR results showed that, compared to TA, the characteristic broad and intense ‐OH peak in the CeTA spectrum was significantly reduced and narrowed. This indicates the participation of hydroxyl groups from TA molecules in coordination, leading to a decrease in free hydroxyl groups. Concurrently, the appearance of vibrational and substitution peaks for the phenyl ring around 1550 and 794 cm^−1^ confirmed the formation of a metal‐polyphenol network (MPN) (Figure 2B). Metal‐phenolic networks (MPNs) are nanoscale complexes formed through coordination bond interactions between metal ions and polyphenolic compounds [54]. The excellent catalytic activity of CeTA is achieved through the reversible redox cycle between Ce^3+^ and Ce^4+^. The surface chemical composition and valence states of CeTA were analyzed by XPS (Figure 2C). The XPS survey spectrum showed characteristic peaks of C, O, and Ce elements, with atomic percentages of 62.77%, 35.38%, and 1.85%, respectively. The contents of Ce^3+^ and Ce^4+^ were 90.94% and 9.06%, respectively, suggesting the presence of predominantly trivalent cerium species. Further validation was performed through morphological and elemental distribution characterization. FE‐TEM and EDS were employed to analyze the morphology and elemental distribution of O and Ce in CeTA. FE‐TEM images revealed irregular morphologies of CeTA (Figure S1A), while combined EDS analysis confirmed the uniform distribution of O and Ce elements (Figure 2D). DLS measurements revealed that the hydrodynamic diameter of CeTA was 58.30 ± 0.58 nm in PBS, 97.28 ± 0.15 nm in FBS, 106.5 ± 1.8 nm in DMEM/F12 basal medium, and 124.9 ± 2.4 nm in deionized water (Figures 2F and S1B–D). In DMEM/F12 basal medium, the hydrodynamic diameter of CeTA gradually increased with prolonged dispersion time (Figure S1E). The zeta potentials were approximately ‐26.26 mV in deionized water, ‐31.07 mV in PBS, −27.90 mV in DMEM/F12 medium, and −31.41 mV in FBS (Figure 2E). Overall, CeTA maintained nanoscale particle sizes without detectable micron‐scale aggregation peaks and exhibited moderate dispersion and stability in physiologically relevant media. Quantitative analysis via ICP‐OES and the Folin‐Phenol reagent method showed that the Ce content in CeTA was 142.690 mg g^−1^, and the TA content was approximately 798.11 mg g^−1^.

Next, we systematically evaluated the multiple enzyme‐like activities of CeTA. The NBT assay, employed to detect SOD‐like activity, demonstrated that CeTA catalyzed the disproportionation of superoxide anion (O_2_•^−^) into H_2_O_2_ and O_2_, with its O_2_•^−^ scavenging capacity increasing in a concentration‐dependent manner (Figure 2G). Further evaluation of CAT‐like activity using a CAT assay kit revealed that CeTA efficiently decomposed H_2_O_2_ to produce O_2_, which also exhibited clear concentration dependence (Figure 2H). Together, these results confirm that CeTA significantly scavenges O_2_•^−^ and H_2_O_2_, demonstrating SOD‐like and CAT‐like enzymatic activities. More importantly, previous studies have demonstrated that the catalytic activity of cerium‐based nanozymes is closely related to the Ce^3+^/Ce^4+^ ratio, with high Ce^3+^ content generally exhibiting stronger SOD activity [55]. In this study, CeTA exists predominantly in the Ce^3+^ form, so it should theoretically exhibit stronger SOD activity. This inference was verified by comparing the SOD and CAT activities of CeTA, CeO_2_, and TA. The SOD activity of CeTA was significantly higher than that of CeO_2_ and TA, with statistically significant differences (Figure 2G). The CAT activity of CeO_2_ was slightly higher than that of CeTA, but the difference between them was not statistically significant. However, the CAT activities of both CeTA and CeO_2_ were significantly higher than that of TA, with statistically significant differences (Figure 2H). Although the initial Ce^4+^ content in CeTA is relatively low, it still exhibits detectable CAT activity, which may be attributed to the oxidation of Ce^3+^ to Ce^4+^ in the presence of H_2_O_2_ [56]. Meanwhile, the polyphenolic structure of TA may also synergistically contribute to H_2_O_2_ scavenging. Therefore, the high proportion of Ce^3+^ in CeTA not only endows it with strong SOD activity, but also enables its dynamic conversion to Ce^4+^ through reaction with H_2_O_2_ to exert CAT activity. This reversible redox cycle endows CeTA with superior antioxidant capacity.

Biocompatibility experiments demonstrated that CeTA exhibited no significant toxicity to mouse bone marrow‐derived mesenchymal stem cells (mBMSCs) at concentrations of 12.5, 25, and 50 µg/mL. Cell viability increased with concentration, and Live/Dead staining revealed minimal cell death. However, at concentrations of 100 and 200 µg/mL, cell viability significantly decreased, indicating dose‐dependent toxicity at high CeTA concentrations (Figure S2A−D). Consequently, 50 µg mL^−1^ was selected as the optimal working concentration of CeTA for subsequent experiments.

### 2.2 Construction and ROS‐Responsive Release of CeTA@GPP Hydrogel

Having established that CeTA possesses robust SOD and CAT like activities with favorable biocompatibility, we next sought to develop a delivery platform capable of retaining CeTA at the injury site and releasing it on demand in response to the pathological microenvironment. To this end, we constructed an injectable ROS‐responsive hydrogel (GPP) based on dynamic boronate ester crosslinking between phenylboronic acid‐modified gelatin (Gel‐PBA) and PVA.

**FIGURE 3:**
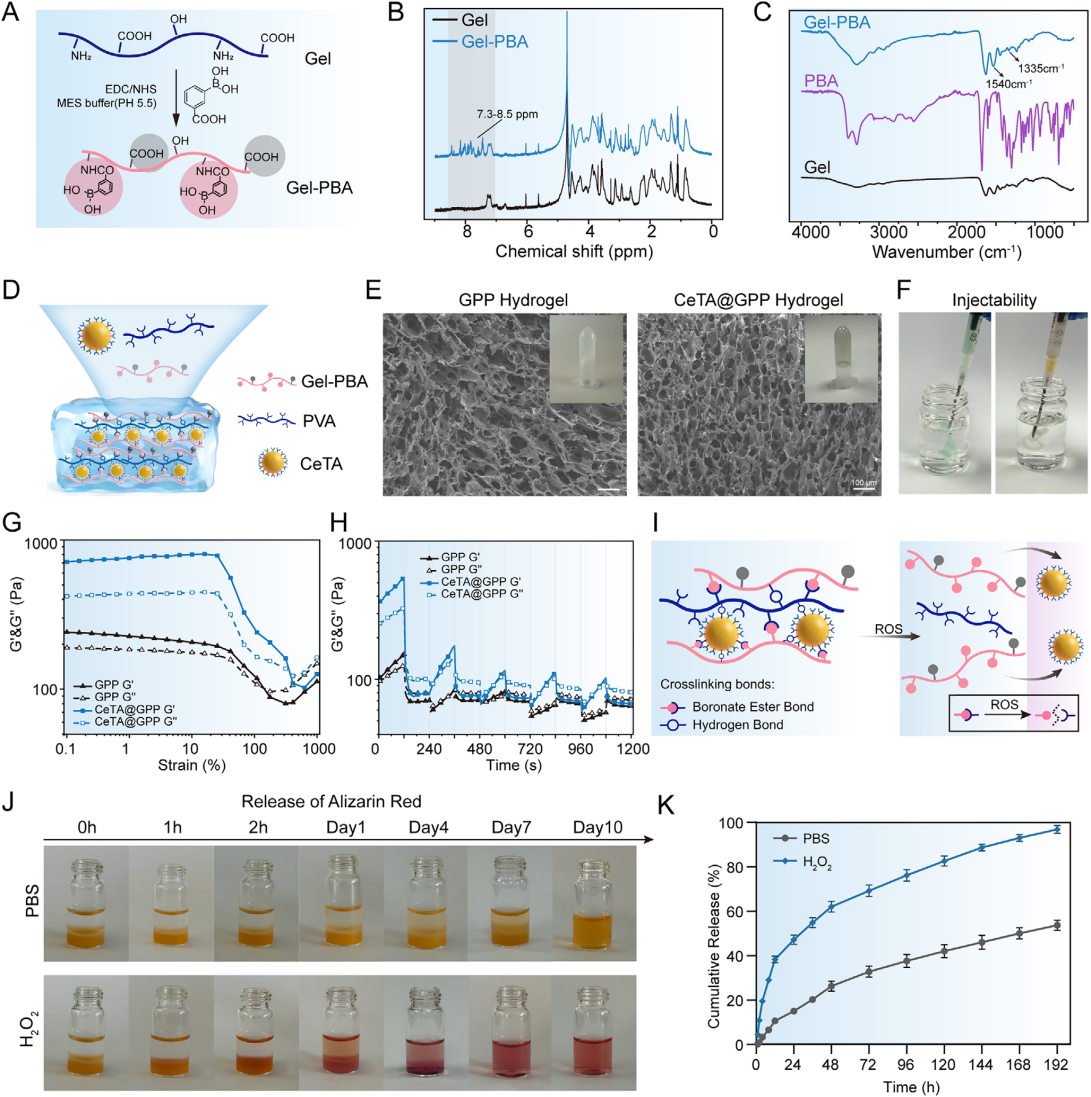
Synthesis and characterization of Gel‐PBA and CeTA@GPP hydrogel. (A) Schematic synthesis of Gel‐PBA. (B) The ^1^H NMR spectra of Gel‐PBA. (C) FTIR patterns of gelatin (gel), PBA and Gel‐PBA. (D) Schematic synthesis of CeTA@GPP hydrogel. (E) Photographs of the hydrogel formation and FE‐SEM images of GPP and CeTA@GPP. Scale bar = 100 µm. (F) Injectability of hydrogel. (G) Strain sweep measurements of G′ and G″ of GPP and CeTA@GPP hydrogel. (H) Alternating strain cycle scanning (1% strain 120 s and 300% strain 120 s) of GPP and CeTA@GPP hydrogel. (I) Schematic illustration of the release mechanism of CeTA from the CeTA@GPP hydrogel system under high ROS conditions. (J) Photographs comparing the changes and degradation behavior of GPP hydrogel (containing alizarin red) in PBS and 0.03% H_2_O_2_ (n = 3). (K) Cumulative release rate (%) of TA from CeTA@GPP hydrogel in PBS or 0.03% H_2_O_2_ (n = 3). All data are expressed as mean ± SD.

In this study, Gel‐PBA was synthesized using the EDC/NHS method (Figure 3A). ^1^H NMR results showed the phenyl proton resonances of Gel‐PBA at 7.3–8.5 ppm (Figure 3B). FTIR analysis showed characteristic stretching vibration peaks of the phenyl ring at 1540 cm^−1^ and the B–O stretching vibration of boronic acid at 1335 cm^−1^ (Figure 3C), confirming the successful synthesis of Gel‐PBA. The injectable GPP hydrogel was prepared by mixing a 6% Gel‐PBA solution with a 5% PVA solution at an equal volume ratio under rapid stirring for cross‐linking. To prepare CeTA@GPP hydrogel, 200 µg/mL CeTA was first mixed with 10% PVA in equal proportions, and then combined with 6% Gel‐PBA solution in equal proportions, followed by stirring to allow crosslinking (Figure 3D). FE‐SEM revealed that the hydrogel possessed a porous, cross‐linked network structure (Figure 3E). EDS analysis further confirmed the uniform distribution of C, O, and Ce elements within the hydrogel scaffold. Furthermore, uniformly distributed C, O, and Ce elements could still be detected in the CeTA@GPP hydrogel after immersion in PBS for 5 days (Figure S4C).

The rheological properties of the hydrogels were evaluated using an advanced rotational rheometer. Time‐sweep analysis revealed that both storage modulus (G′) and loss modulus (G″) increased with time for both hydrogels. CeTA@GPP hydrogel exhibited a higher G′ (approximately 420 Pa) compared to GPP (approximately 238 Pa), indicating that CeTA enhanced the compactness and crosslinking density of the hydrogel network (Figure S3A). Frequency sweeps showed that G′ progressively exceeded G″, indicating a semi‐rigid nature. The incorporation of CeTA increased the modulus, forming a more stable crosslinked network (Figure S3B). Strain‐sweep tests revealed that both the initial G′ (approximately 670 Pa) and G″ (approximately 438 Pa) of CeTA@GPP hydrogel were higher than those of GPP (G′: approximately 263 Pa, G″: approximately 197 Pa). As strain increased, CeTA@GPP hydrogel exhibited G″ > G′ at approximately 400% strain, whereas GPP showed G″ > G′ at around 100% strain, indicating that CeTA delayed the elastic‐to‐viscous transition (Figure 3G). Step‐strain cycling (1% strain and 300% strain) revealed that G′ decreased and became smaller than G″ at 300% strain, confirming the gel‐sol transition. Due to the dynamic reversibility of boronate ester bonds, G′ rapidly recovered upon the removal of high strain, demonstrating the hydrogel's self‐healing properties (Figure 3H). Both GPP and CeTA@GPP hydrogels could be delivered via syringe injection (Figure 3F), enabling long‐term coverage of uterine injury sites while maintaining structural integrity in dynamic environments.

Previous studies have shown that the concentration of H_2_O_2_ in exudates from the inflammatory microenvironment gradually increases with the severity of infection [57]. The 0.03% H_2_O_2_ used in this study falls within this range. The ROS responsiveness of the hydrogel was assessed at this concentration. Alizarin red‐GPP hydrogel was placed in PBS and 0.03% H_2_O_2_ solutions, respectively. In the 0.03% H_2_O_2_ group, a color change was initiated at 1 h and transitioned to orange‐red by 2 h. The hydrogel in 0.03% H_2_O_2_ underwent complete dissolution by day 7, whereas complete dissolution in PBS required 14 days (Figure 3I,J). In vitro degradation experiments further confirmed this observation. In PBS, the degradation rate of the CeTA@GPP hydrogel was significantly lower than that in the H_2_O_2_ group, with degradation beginning to accelerate on day 5 and not completing until day 14. In contrast, in H_2_O_2_, the hydrogel was completely degraded by day 7 (Figure S4A). Furthermore, FE‐SEM showed that after immersion in PBS or H_2_O_2_ for 1, 3, and 5 days, both groups of hydrogels retained a three‐dimensional porous network structure. However, with prolonged incubation time, the pores in the H_2_O_2_ group were significantly larger than those in the PBS group, indicating that H_2_O_2_ accelerates the collapse of the CeTA@GPP hydrogel backbone network (Figure S4B). Collectively, these results confirmed the ROS‐responsive degradation of the CeTA@GPP hydrogel. TA release kinetics showed that the cumulative release rate reached 54.86% at 36 h and 96.87% by day 8 in 0.03% H_2_O_2_. In contrast, the cumulative release rate in PBS was only 53.67% at day 8 (Figure 3K).

The above experiments confirmed that the hydrogel system exhibits sustained‐release capability and achieves targeted, on‐demand release triggered by high‐ROS inflammatory microenvironments. Furthermore, CCK‐8 assays and Live/Dead staining demonstrated that both the GPP and CeTA@GPP hydrogels possess excellent biocompatibility and no cytotoxicity toward mBMSCs (Figure S5A–D).

### 2.3 CeTA@GPP Hydrogel Modulates Macrophage Polarization and Alleviates Oxidative Stress

The above results confirmed that CeTA@GPP hydrogel achieves ROS‐triggered on demand release with excellent biocompatibility. Since the injured endometrium is characterized by M1 macrophage dominated inflammation and excessive ROS accumulation, we next investigated whether CeTA@GPP hydrogel could modulate macrophage polarization and alleviate oxidative stress in vitro.

We employed a combination of lipopolysaccharide (LPS) and interferon‐gamma (IFN‐γ) stimulation to model the inflammatory microenvironment following endometrial injury, thereby promoting the M1 polarization of macrophages. Laser scanning confocal microscopy was used to observe the cellular uptake of Cy5.5‐labeled CeTA by RAW264.7 cells at 24 and 48 h. The results demonstrated that M1‐polarized RAW264.7 cells could effectively internalize Cy5.5‐labeled CeTA (Figure S6). Notably, fluorescently tagged Cerium oxide nanoparticles have previously been shown to be governed by energy‐dependent, clathrin‐mediated, and caveolae‐mediated endocytic pathways [58].

**FIGURE 4:**
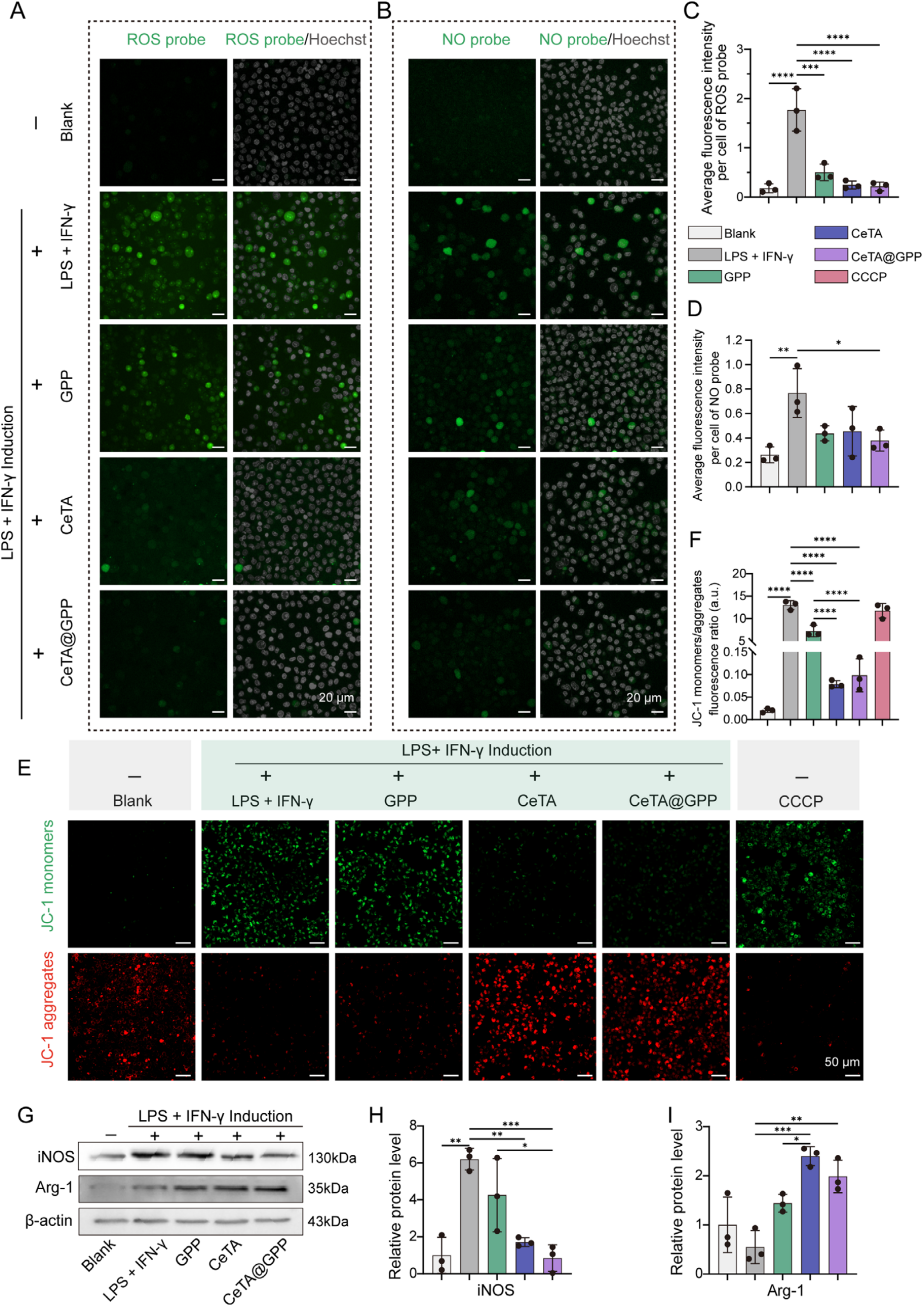
The effects of CeTA@GPP hydrogel on RAW264.7 in vitro. (A–D) Immunofluorescence (IF) microscopy images showed ROS probe and NO probe expression (green fluorescence) in macrophages under different treatment conditions. Nuclei were stained with Hoechst 33342 (grey). Scale bar: 20 µm. The statistical results for the average fluorescence intensity of the ROS probe and NO probe in each cell are shown (n = 3). (E,F) IF microscopy images showed the expression of JC‐1 monomers (green) and JC‐1 aggregates (red) in macrophages under different treatment conditions, with quantification of the fluorescence intensity ratio of JC‐1 monomers to JC‐1 aggregates. (G‐I) WB was used to assess the protein expression of iNOS and Arg‐1. The representative WB images and statistical results are shown (n = 3). All data are expressed as mean ± SD (*p < 0.05, **p < 0.01, ***p < 0.001).

The inflammatory microenvironment within the uterine cavity induces M1 polarization of macrophages. This process involves the upregulation of iNOS, which catalyzes the production of excessive nitric oxide (NO) from L‐arginine, and activation of enzymes such as NADPH oxidase, leading to ROS generation [59]. Excessive ROS disrupts the mitochondrial electron transport chain, causing a decline in mitochondrial membrane potential and subsequently inducing energy metabolism disorders and apoptosis [60]. Therefore, NO, ROS, and mitochondrial membrane potential serve as key biomarkers for pro‐inflammatory responses, oxidative stress, and mitochondrial functional integrity, respectively. In this study, changes in intracellular NO and ROS levels in RAW264.7 were quantitatively detected using the DAF‐FM DA and DCFH‐DA probes, respectively. The results showed no significant difference in NO or ROS levels between the GPP group and the LPS + IFN‐γ group. However, after a 24 h treatment with CeTA or the CeTA@GPP hydrogel, the fluorescence intensities of both probes in M1‐polarized macrophages were markedly reduced compared with those in the LPS + IFN‐γ group and the GPP group (Figure 4A–D). Mitochondrial membrane potential was subsequently assessed using the JC‐1 fluorescent probe. The results revealed a marked increase in the ratio of JC‐1 monomer to polymer in the LPS + IFN‐γ and GPP groups, indicating a significant decline in mitochondrial membrane potential and mitochondrial damage. In contrast, after 24 h of treatment with CeTA or the CeTA@GPP hydrogel, the JC‐1 monomer/polymer ratio in M1 macrophages showed no significant difference compared to the blank group, suggesting that the mitochondrial membrane potential was restored to normal levels following intervention (Figure 4E,F).

Western blot (WB) analysis was performed to detect the M1 marker inducible nitric oxide synthase (iNOS) and the M2 marker arginase‐1 (Arg‐1). The results showed that LPS and IFN‐γ stimulation significantly increased iNOS expression in RAW264.7 cells, while treatment with CeTA or CeTA@GPP hydrogel for 24 h markedly decreased it. Concurrently, Arg‐1 expression was significantly upregulated in RAW264.7 cells following CeTA and CeTA@GPP hydrogel treatment. In contrast, no statistically significant differences in iNOS or Arg‐1 expression were observed between the GPP group and the LPS + IFN‐γ group (Figure 4G–I). Similar phenomena have also been reported in previous literature [43, 61]. Combining previous studies with the experimental results of this work, it can be reasonably inferred that the immunomodulatory effect of the CeTA@GPP hydrogel mainly originates from the loaded CeTA. These findings confirmed that CeTA and the CeTA@GPP hydrogel significantly inhibited M1 polarization in macrophages and simultaneously exerted anti‐inflammatory effects.

The in vitro results from RAW264.7 cells demonstrated that the CeTA@GPP hydrogel possesses anti‐inflammatory and antioxidant properties, protects mitochondrial function, and inhibits M1 macrophage polarization. These actions collectively contribute to establishing a local microenvironment conducive to endometrial regeneration.

### 2.4 CeTA@GPP Hydrogel Reverses TGF‐β1‐Induced Fibrosis in HESCs

**FIGURE 5:**
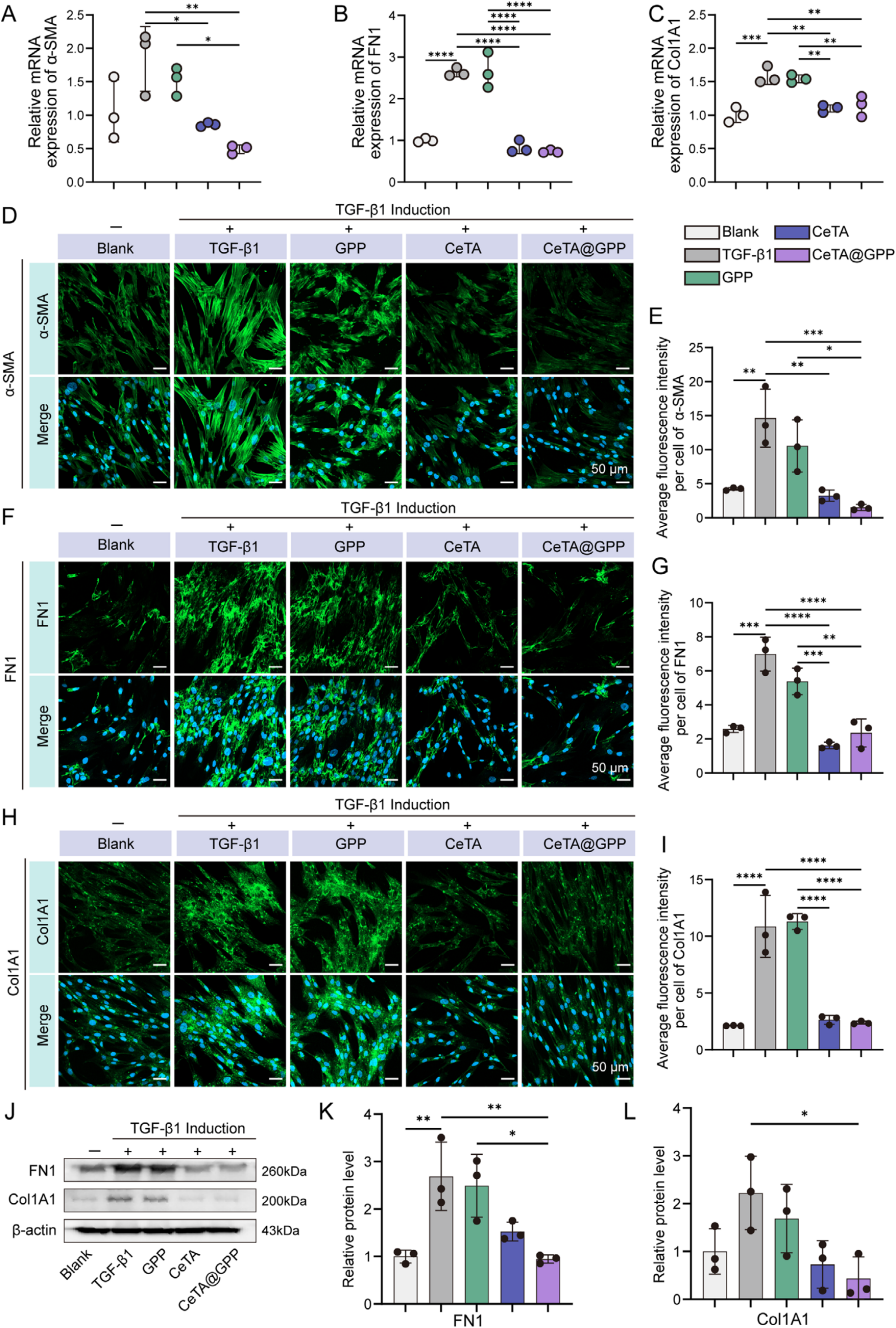
The anti‐fibrotic effects of CeTA@GPP hydrogel on hESCs in vitro. (A–C) The mRNA expression levels of α‐SMA, FN1, and Col1A1 was assessed using RT‐qPCR, with GAPDH or β‐actin as a normalization control (n = 3). (D–I) The average fluorescence intensity of α‐SMA, FN1, and Col1A1 in each cell was evaluated by IF staining in each group (n = 3). Nuclei were stained with DAPI (blue). Scale bar: 50 µm. (J–L) WB was used to assess the protein expression of FN1 and Col1A1. The representative WB images and statistical results are shown (n = 3). All data are expressed as mean ± SD (*p < 0.05, **p < 0.01, ***p < 0.001, ****p < 0.0001).

The above findings demonstrated that CeTA@GPP hydrogel effectively suppresses M1 polarization and restores redox homeostasis. Given that unresolved inflammation and oxidative stress directly drive fibroblast‐to‐myofibroblast transformation and excessive ECM deposition in IUA, we further examined whether CeTA@GPP hydrogel could reverse TGF‐β1‐induced fibrosis in hESCs. To model endometrial fibrosis in vitro, hESCs were stimulated with TGF‐β1, followed by 24 h treatment with GPP extract, CeTA, or CeTA@GPP hydrogel, respectively. Fibrotic markers associated with myofibroblast activation and ECM deposition, including α‐SMA, Col1A1, and FN1, were assessed by RT‐qPCR, IF staining, and WB analyses. TGF‐β1 stimulation significantly upregulated the mRNA levels of α‐SMA, Col1A1, and FN1, confirming successful fibrosis induction. Treatment with CeTA or CeTA@GPP hydrogel restored these markers to levels comparable to the blank group, whereas GPP alone showed no significant effect (Figure 5A–C). These trends were corroborated at the protein level by IF staining (Figure 5D–I) and WB analysis of Col1A1 and FN1 (Figure 5J–L). Collectively, these results demonstrated that the anti‐fibrotic activity of CeTA@GPP hydrogel is attributable to CeTA rather than the hydrogel matrix itself.

Additionally, we performed a cell scratch assay to simulate and evaluate the wound healing in vitro process using the hESCs as a model. Apparently, the CeTA@GPP hydrogel group had the highest cell migration efficacy among the five groups (Figure S7A–D). After 12 h, the migration rate in the TGF‐β1 group was only 3.9%, while GPP, CeTA, and CeTA@GPP hydrogel increased migration to approximately 1.27‐fold, 1.96‐fold, and 2.86‐fold relative to the blank group, respectively. After 24 h, both TGF‑β1 and GPP remained below the level of the blank group, whereas CeTA and CeTA@GPP hydrogel significantly increased migration to about 1.62 fold and 1.91 fold of the blank group. After 36 h, GPP approached and slightly surpassed the blank group and was markedly higher than TGF‑β1, while CeTA and CeTA@GPP hydrogel promoted migration to more than 1.5‐fold and 1.7 fold of the blank group, respectively. In summary, CeTA and CeTA@GPP hydrogel significantly enhanced hESCs migration capacity, thereby facilitating the healing of uterine cavity injuries.

Based on the above in vitro results, we can preliminarily conclude that CeTA and CeTA@GPP hydrogel inhibit M1 polarization of macrophages and reduce intracellular NO and ROS levels. By modulating the inflammatory microenvironment, these effects create a local environment favorable for endometrial regeneration. Furthermore, CeTA and the CeTA@GPP hydrogel significantly inhibit the transformation of hESCs into myofibroblasts and suppress excessive ECM deposition. Collectively, these effects effectively reverse hESCs fibrosis and may contribute to the prevention and treatment of IUA.

### 2.5 CeTA@GPP Hydrogel Inhibits Intrauterine Adhesion Formation in Rat Model

**FIGURE 6:**
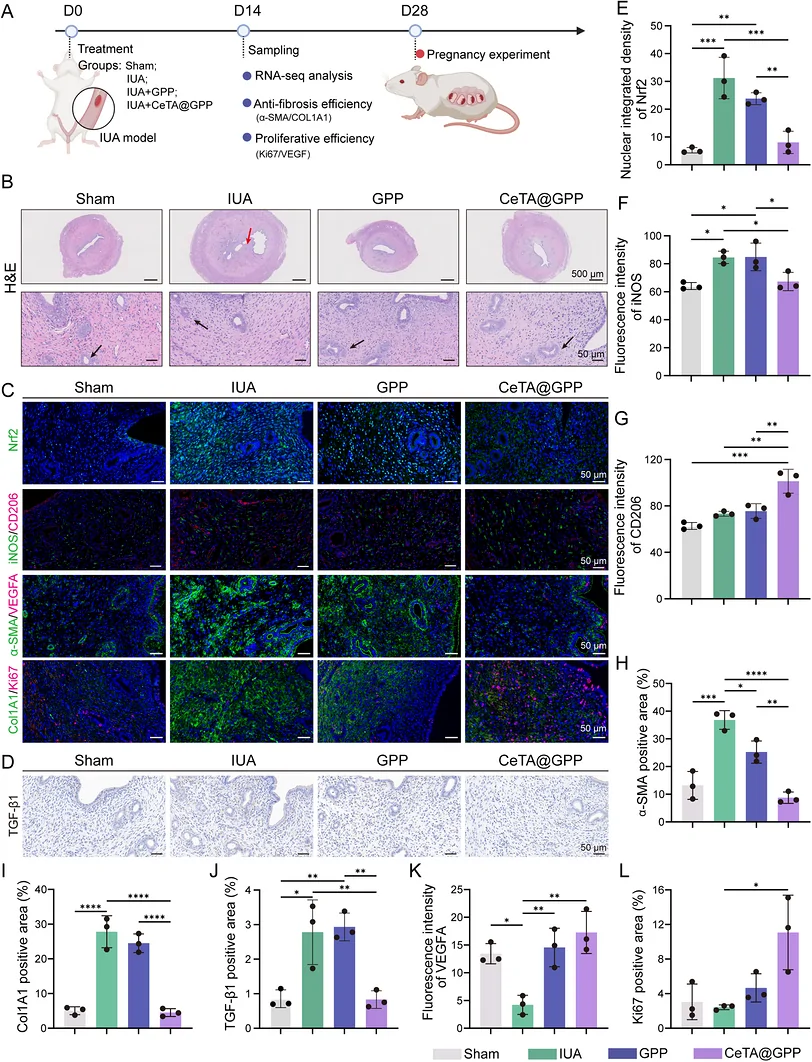
In vivo effects of CeTA@GPP hydrogel in endometrial repair. (A) The schematic illustration of in vivo IUA model construction, treatment after endometrial damage, and subsequent experimental procedures. (B) Representative images of H&E staining of rat uteri sections at 14 days postoperatively. (C, E–I, K,L) IF staining for Nrf2, iNOS, CD206, α‐SMA, VEGFA, Col1A1, and Ki67 in uterine tissues. The representative IF staining images and statistical results are shown (n = 3). (D, J) Representative immunohistochemical staining images and quantification of TGF‐β1 (n = 3). All data are expressed as mean ± SD (*p < 0.05, **p < 0.01, ***p < 0.001, ****p < 0.0001).

Our in vitro studies collectively demonstrated that CeTA@GPP hydrogel modulates the inflammatory microenvironment, scavenges ROS, and inhibits fibrotic transformation of hESCs. To validate these therapeutic effects in a physiologically relevant context, we established a rat IUA model using combined mechanical injury and LPS infection to evaluate the in vivo efficacy of CeTA@GPP hydrogel (Figure 6A).

Dynamic boronate ester bond‐crosslinked systems based on Gel‐PBA and PVA have been used in studies of cutaneous wound repair, including diabetic wound healing [62] and infected skin injury [63], suggesting that this type of hydrogel possesses good tissue adaptability and local coverage potential. In this study, in vivo imaging was performed on rats injected with CeTA‐Cy5.5@GPP on days 1, 3, 6, and 14 post‐surgery. The results showed a distinct fluorescent signal in the uterine region on day 1 after intrauterine injection. The signal persisted within the uterus on day 3, though it gradually weakened. On day 6, the fluorescence was primarily localized in the lower uterine segment, and at day 14, the signal had significantly diminished (Figure S8A). These findings confirmed that the CeTA@GPP hydrogel can achieve prolonged residence and retain its efficacy at the site of injury.

On day 14 post‐operation, uteri from each group were collected and photographed to document their morphology (Figure S8B). Notably, uteri in both the IUA group and the GPP group exhibited significant swelling with the presence of yellow exudate within the lumen. In contrast, the uterine morphology was partially restored in rats treated with the CeTA@GPP hydrogel. H&E staining revealed poor endometrial regeneration in the IUA group (Figure 6B). Quantitative analysis showed that endometrial thickness was significantly reduced and the number of glands was markedly decreased in the IUA group compared to the other three groups. However, no statistically significant differences were observed in either endometrial thickness or gland number between the GPP group, the CeTA@GPP group, and the Sham group (Figure S8D,E).

To further investigate the in vivo antioxidant effect of the CeTA@GPP hydrogel, we examined the expression of Nrf2 and the activities of the core antioxidant enzymes SOD and CAT in uterine tissue. Nrf2 is a key transcription factor in the antioxidant defense system. IF staining for Nrf2 showed that (Figure 6C,E), compared with the Sham group, Nrf2 expression was significantly upregulated in the IUA and GPP groups. Compared with the IUA and GPP groups, CeTA@GPP hydrogel intervention significantly downregulated Nrf2 expression, although its expression remained slightly higher than that in the Sham group. The results of SOD and CAT activity assays in uterine tissue showed that (Figure S9A–C), compared with the Sham group, SOD and CAT activities in the IUA group were significantly decreased. This result indicates that the onset of IUA is accompanied by a sharp elevation of oxidative stress levels in the endometrium, leading to severe impairment of the antioxidant defense system. The changes in SOD and CAT activities in the IUA group are consistent with previous IUA studies [64, 65]. Meanwhile, treatment with GPP hydrogel alone showed limited recovery of SOD and CAT activities. In contrast, CeTA@GPP hydrogel intervention significantly restored SOD and CAT activities in uterine tissue. These results indicate that the endometrium in IUA is under severe oxidative stress, with the endogenous antioxidant system being compensatorily activated. The CeTA@GPP hydrogel directly scavenges excess ROS in the pathological uterine microenvironment, thereby alleviating the compensatory activation of the endogenous antioxidant system. This finding strongly supports that the antioxidant effect of the CeTA@GPP hydrogel is primarily attributable to its direct ROS‐scavenging capability, and that it can effectively restore the ability of uterine tissue to scavenge O_2_•^−^ and H_2_O_2_ in vivo.

To evaluate the regulatory effect of the CeTA@GPP hydrogel on macrophage polarization in vivo, we examined the expression of the M1 macrophage marker iNOS and the M2 macrophage marker CD206. IF staining for iNOS showed that iNOS expression was significantly upregulated in the IUA and GPP groups compared with the Sham group, while CeTA@GPP hydrogel intervention significantly prevented this upregulation (Figure 6C,F). Meanwhile, IF staining for CD206 revealed that CeTA@GPP hydrogel intervention significantly upregulated CD206 expression compared with the other three groups (Figure 6C,G). These results indicate that the CeTA@GPP hydrogel can effectively inhibit macrophage polarization toward the pro‐inflammatory M1 phenotype while promoting polarization toward the anti‐inflammatory and pro‐repair M2 phenotype in vivo.

The TGF‐β signaling pathway is a key fibrosis‐related signaling pathway. Immunohistochemical staining for TGF‐β1 showed that, compared with the Sham group, its expression was significantly upregulated in the IUA and GPP groups. Compared with the IUA and GPP groups, CeTA@GPP hydrogel intervention significantly downregulated its expression (Figure 6D,J). During the repair process of the injured endometrium, fibroblasts undergo abnormal activation and transform into myofibroblasts, excessively secreting and depositing ECM components such as Col1A1, thereby impeding glandular regeneration and vascular reconstruction [66]. α‐SMA serves as a marker for fibroblast‐to‐myofibroblast conversion. In this study, the protein expression levels of α‐SMA and Col1A1 in endometrial tissue sections were assessed via IF staining. The results showed significantly enhanced fluorescence intensity of both α‐SMA and Col1A1 in the IUA group, indicating widespread myofibroblast activation and excessive collagen accumulation in the model group. Compared with the IUA group, both the GPP and CeTA@GPP groups exhibited varying degrees of downregulation in Col1A1 and α‐SMA protein levels within the endometrium. Notably, the expression levels in the CeTA@GPP group were restored to levels comparable to those in the Sham group (Figure 6C,H,I). Masson trichrome staining (Figure S8C) indicated increased collagen fiber deposition in both the IUA and GPP groups. Conversely, collagen deposition was significantly reduced in the CeTA@GPP group, approaching those of the Sham group (Figure S8F). Collectively, these results further confirmed that the CeTA@GPP hydrogel can effectively inhibit the fibrotic process driven by the TGF‐β1 signaling pathway in vivo, including blocking myofibroblast transformation and reducing excessive Col1A1 deposition.

Endometrial tissue reconstruction relies on the proliferation and angiogenesis of endometrial epithelial cells and stromal cells [67, 68]. Ki67, a marker for cellular proliferation activity, reflects the level of cellular proliferation [69]. VEGFA, a core regulatory factor in angiogenesis, facilitates the reconstruction of the functional capillary network in the endometrium [70]. IF analysis revealed the lowest Ki67 fluorescence signal in the IUA group, indicating that the pathological state suppressed endometrial cell proliferation. Compared to the other three groups, the CeTA@GPP hydrogel treatment significantly upregulated nuclear Ki67 fluorescence signal, which was markedly higher than that in both the Sham and IUA groups (Figure 6C,L). This suggested that the hydrogel intervention enhanced endometrial proliferation, thereby promoting endometrial repair. Similarly, the IUA group exhibited the lowest VEGFA‐positive signal, indicating poor angiogenic potential. In contrast, the CeTA@GPP group showed the highest VEGFA fluorescence signal. Compared to the IUA group, VEGFA protein expression in both the GPP and CeTA@GPP groups was restored to levels observed in the Sham group, with the CeTA@GPP group demonstrating the highest degree of upregulation (Figure 6C,K). Previous studies have confirmed that nanoceria can induce angiogenesis by modulating the intracellular oxygen environment and stabilizing HIF‐1α, and its pro‐angiogenic activity is closely correlated with the Ce^3+^/Ce^4+^ ratio [71]. This is highly consistent with the experimental results of the present study. These findings demonstrated that the CeTA@GPP hydrogel promotes the proliferation and differentiation of endometrial cells by enhancing angiogenesis, thereby facilitating the organized regeneration of the functional layer of the endometrium following injury.

In summary, in the IUA rat model, the GPP hydrogel functions as an in situ physical barrier that separates the injured uterine walls and provides a temporary scaffold for re‐epithelialization. This barrier effect contributes to restoring the gross structure of the endometrium [72]. In contrast, blocking the processes of inflammation, oxidative stress, and fibrosis primarily depends on the biological activity of CeTA. The synergistic action of the two enables the CeTA@GPP hydrogel to effectively scavenge excess ROS, regulate macrophage polarization toward the M2 phenotype, inhibit fibrosis, and promote cell proliferation and angiogenesis, thereby facilitating the structural and functional repair of the damaged endometrium.

### 2.6 Transcriptomic Analysis Reveals Multimechanistic Actions of CeTA@GPP Hydrogel

**FIGURE 7:**
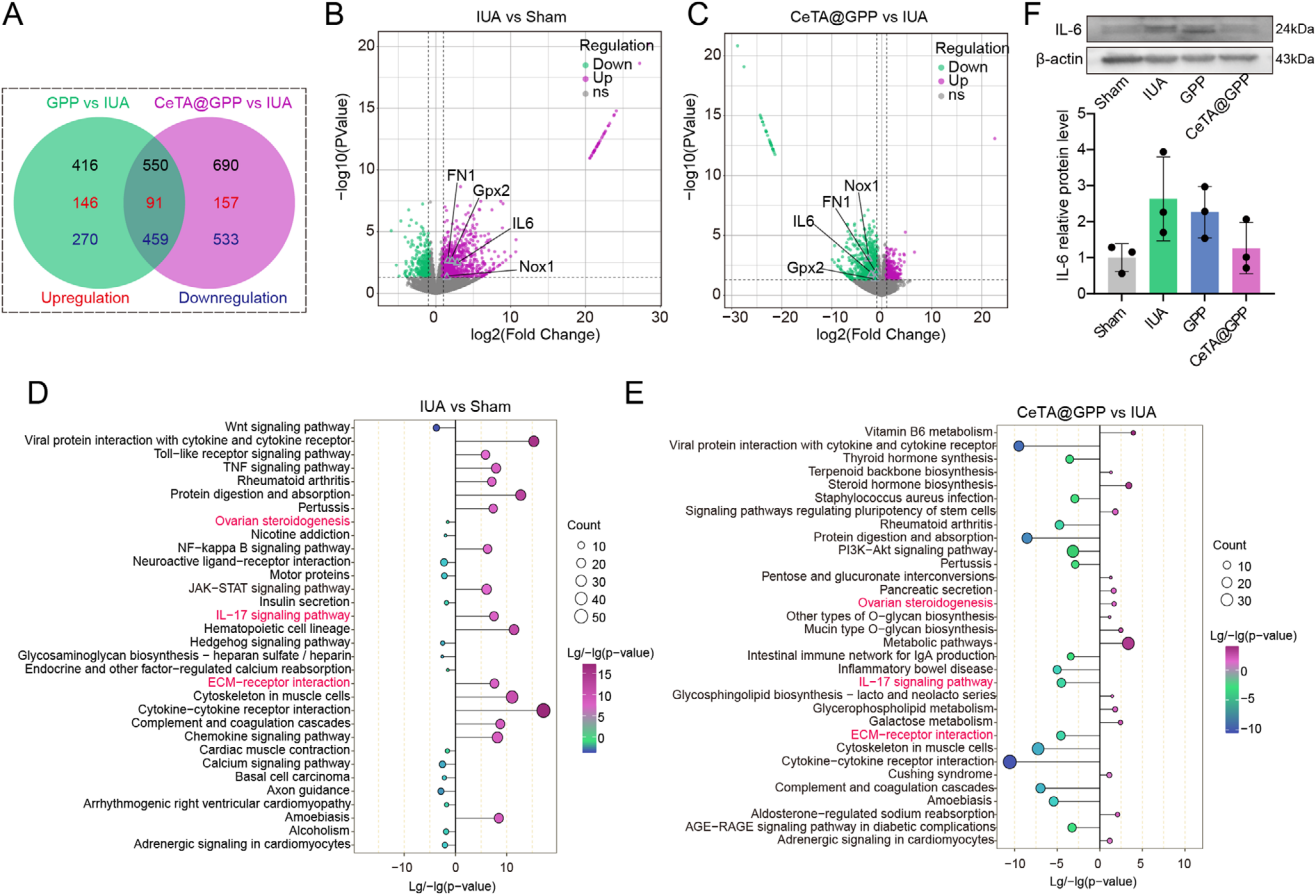
Transcriptomic profiling of endometrial tissue across all groups (n = 3). (A) Venn diagram of DEGs (|log_2_FoldChange|≥1, *p* value < 0.05) between the GPP group and the IUA group and between the CeTA@GPP group and the IUA group. (B,C) Volcano plot of DEGs in IUA vs. Sham and CeTA@GPP vs. IUA. (D,E) KEGG pathway enrichment of IUA vs. Sham and CeTA@GPP vs. IUA. Bubble size: gene count. Color: Rich factor (Left side of the x‐axis: downregulated DEGs enrichment pathways; right side of the x‐axis: upregulated DEGs enrichment pathways). (F) WB results of IL‐6 (n = 3). All data are expressed as mean ± SD.

The histological and IF results above demonstrated that CeTA@GPP hydrogel alleviates fibrosis and promotes endometrial regeneration in vivo. To gain mechanistic insight into these therapeutic effects at the molecular level, we performed transcriptomic profiling of endometrial tissues across all groups. The results showed that, compared to the IUA group, 237 genes were significantly upregulated and 729 genes were significantly downregulated in the GPP group. Similarly, 248 upregulated and 992 downregulated genes were identified in the CeTA@GPP group versus the IUA group. Among these DEGs, a total of 550 genes overlapped, including 91 commonly upregulated genes and 459 commonly downregulated genes (Figure 7A).

To further evaluate the anti‐inflammatory, antioxidant, and anti‐fibrotic effects of the CeTA@GPP hydrogel at the transcriptional level and to assess their consistency with the protein‐level findings, we analyzed the expression changes of the relevant genes. The volcano plots visually present the changes in gene expression between groups. Compared with the Sham group, volcano plots revealed significantly upregulated expression levels of interleukin‐6 (IL‐6), NADPH oxidase 1 (NOX1), glutathione peroxidase 2 (GPX2), and FN1 in the IUA group (Figure 7B). IUA involves a pathological network of inflammation, oxidative stress, and fibrosis. Concurrently, increased FN1 expression reflects ECM deposition, exacerbating fibrosis. Compared to the IUA group, the GPP group downregulated the expression of NOX1 (Figure S10A), while the CeTA@GPP group significantly downregulated expression of IL‐6, NOX1, GPX2, and FN1 simultaneously (Figure 7C). Furthermore, compared with the GPP group, the CeTA@GPP group significantly downregulated IL‐6 expression (Figure S10B), indicating that the incorporation of CeTA further enhanced the anti‐inflammatory effect. More importantly, there were no statistically significant differences in the mRNA expression levels of IL‐6, CD86, GPX2, and FN1 between the Sham group and the CeTA@GPP group (Figure S10C), while Arg‐1 expression was significantly upregulated, which corroborated the expression trend of Arg‐1 in vitro and the IF results of CD206 in vivo. Among the four groups, the pro‐inflammatory genes (CD80, IL‐6) were significantly downregulated in the CeTA@GPP group (Figure S11A), the oxidative stress related gene NOX1 was significantly downregulated, the antioxidant gene NQO1 was significantly upregulated (Figure S11B), and the fibrosis related gene FN1 was also significantly downregulated (Figure S11C). Notably, the downregulation of IL‐6 suggests that the CeTA@GPP hydrogel may inhibit the fibrotic process through modulation of the JAK‐STAT signaling pathway, and WB analysis further confirmed that IL‐6 protein expression exhibited a downward trend in this group (Figure 7F).

KEGG pathway enrichment analysis showed that inflammatory and fibrotic pathways (TNF, IL‐17, NF‐κB signaling pathways, and ECM‐receptor interaction) were significantly activated in the IUA group, while tissue repair‐related pathways (ovarian steroidogenesis) were suppressed (Figure 7D). In contrast, the CeTA@GPP group upregulated signaling pathways such as steroid hormone biosynthesis and ovarian steroidogenesis, and downregulated inflammatory and fibrotic pathways including the IL‐17 signaling pathway and ECM‐receptor interaction (Figure 7E). Furthermore, compared with the GPP group, the downregulated genes in the CeTA@GPP group were enriched in inflammatory pathways such as TNF, IL‐17, and NF‐κB (Figure S10D). These KEGG pathway enrichment analysis results confirmed the effectiveness of CeTA@GPP hydrogel intervention.

GO functional enrichment analysis revealed key biological features associated with CeTA@GPP hydrogel treatment. At the Biological Process level, the DEGs were enriched in processes such as regulation of multicellular organismal processes, cell surface receptor signaling pathways, and cell adhesion, suggesting that the therapeutic intervention may broadly impact intercellular communication and cell‐microenvironment interactions. At the Cellular Component level, the DEGs were primarily localized to the extracellular region, extracellular matrix, and collagen‐containing extracellular matrix, suggesting that the treatment may influence extracellular microenvironment remodeling. At the Molecular Function level, the DEGs were enriched in signaling receptor binding, protein binding, and extracellular matrix structural constituent (Figure S10E).

PPI network analysis highlighted a core network centered on immune regulation while also involving ECM remodeling (Figure S12).

The DEGs analysis and enrichment analysis results suggest that the CeTA@GPP hydrogel can simultaneously regulate the expression of genes related to inflammation, oxidative stress, and fibrosis at the transcriptional level, and achieve multi‐pathway synergistic intervention.

### 2.7 CeTA@GPP Hydrogel Promotes Functional Recovery and Fertility Restoration

**FIGURE 8:**
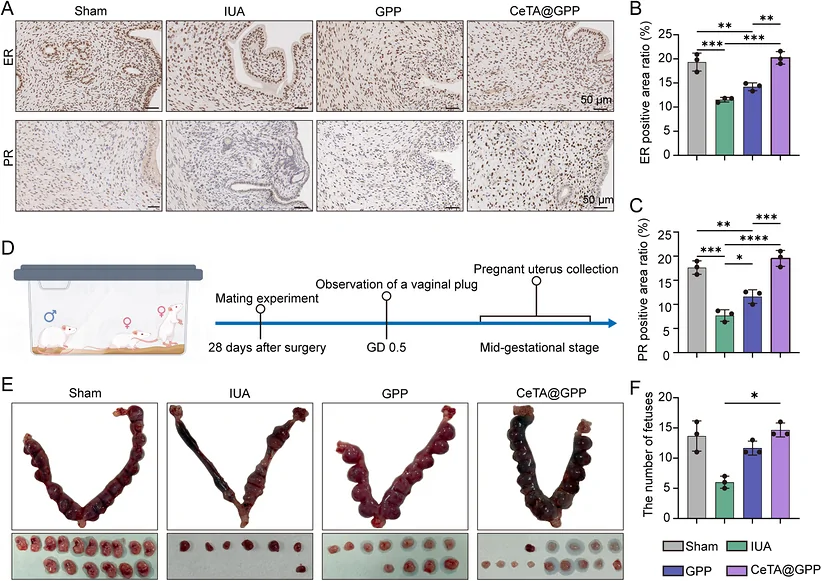
The effects of CeTA@GPP hydrogel on uterine receptivity and fertility. (A–C) Representative immunohistochemical staining images and quantification of ER and PR as markers of endometrial receptivity (n = 3). (D) Experimental timeline for fertility assessment: mating was initiated 28 days post‐treatment. Pregnant uterine samples were collected during mid‐gestation. (E) Representative photographs of pregnant uteri in each group and their corresponding embryonic photographs (n = 3). (F) Statistical analysis of fetal numbers per group (n = 3). All data are expressed as mean ± SD (*p < 0.05).

Transcriptomic analysis confirmed that CeTA@GPP hydrogel modulates inflammatory, oxidative stress, and fibrotic pathways while upregulating steroid hormone biosynthesis. Since endometrial receptivity and fertility restoration are the ultimate clinical endpoints for IUA treatment, we finally assessed whether these molecular and structural improvements translated into functional recovery. As key indicators of endometrial receptivity, ER [73] and PR [74] protein expression in the uterus was evaluated via immunohistochemical staining two weeks post‐operation [75]. The results showed that ER expression in the CeTA@GPP group was significantly higher than that in both the IUA and GPP groups, returning to a level comparable to the Sham group (Figure 8A,B). Concurrently, PR expression levels in the GPP and CeTA@GPP groups were significantly higher than those in the IUA group. Notably, after CeTA@GPP hydrogel treatment, no significant difference in PR expression was observed between the CeTA@GPP group and the Sham group (Figure 8A,C), indicating that CeTA@GPP hydrogel treatment improved endometrial receptivity.

Subsequently, we examined the recovery of fertility in rats with uterine injuries through pregnancy experiments. Rats were mated 28 days post‐surgery, with the observation of a vaginal plug designated as gestational day (GD) 0.5. Uteri were harvested during the mid‐gestational stage for embryo counting (Figure 8D). Statistical analysis revealed that, compared to the IUA group (6 embryos), the number of embryos was significantly increased in both the GPP group (11.7 embryos) and the CeTA@GPP group (14.7 embryos). The embryo counts in these treatment groups were comparable to the Sham group (13.7 embryos). Notably, the CeTA@GPP group exhibited a significant difference in embryo count compared to the IUA group (Figure 8E,F), confirming the significant efficacy of CeTA@GPP hydrogel in restoring rat fertility. This study assessed fertility recovery based on mid‐gestation embryo counts, and future studies could further incorporate indicators such as live birth rates and monitoring of pup growth and development for a more comprehensive evaluation.

Furthermore, to assess the overall biological safety of CeTA@GPP hydrogel treatment at the organismal level, we performed H&E staining on major organs, including the heart, liver, spleen, lung, and kidney. The results revealed no evident organ damage or toxicity (Figure S13). In addition, we further measured serum alanine transaminase (ALT) and aspartate transaminase (AST) in rats of each group to evaluate potential liver injury, and detected serum creatinine (CREA) to assess renal function. The results showed that the levels of ALT, AST, and CREA in all groups were within the normal range, with no significant differences among groups (Figure S14). In addition, no significant differences were observed in body weight changes among the four groups, further supporting that treatment with this hydrogel system did not induce systemic toxicity and exhibited good in vivo biocompatibility (Figure S15). The biocompatibility of the cerium‐tannic acid coordination system has been systematically validated in multiple studies covering various disease models, including myocardial ischemia‐reperfusion injury, acute kidney injury, viral pneumonia, and diabetic osseointegration. Across these models, the system did not induce any significant organ toxicity or tissue damage, but instead demonstrated favorable therapeutic and protective effects [40, 41, 46, 47]. Collectively, these findings demonstrated that CeTA@GPP hydrogel synergistic therapy promotes functional endometrial regeneration and successfully restores fertility. In future studies, longer‐term animal follow‐up experiments will be needed to investigate the long‐term risk of IUA recurrence after complete degradation of the hydrogel.

## 3 Conclusion

In summary, the CeTA@GPP hydrogel harnesses the pathological oxidative microenvironment as an endogenous trigger for therapeutic release. Through boronate ester chemistry, the hydrogel achieves spatiotemporal control over nanozyme delivery, exhibiting dramatically accelerated release kinetics in oxidative environments. This stimulus‐responsive behavior ensures preferential drug accumulation at sites of maximal inflammation‐driven ROS generation.

At the molecular level, CeTA nanozymes exhibit concentration‐dependent SOD‐like and CAT‐like activities that efficiently eliminate superoxide radicals and hydrogen peroxide. These antioxidant actions restore cellular redox homeostasis and mitochondrial membrane potential, thereby protecting endometrial cells from oxidative damage. At the cellular level, CeTA@GPP hydrogel orchestrates immune remodeling by promoting macrophage polarization from pro‐inflammatory M1 to pro‐reparative M2 phenotypes, while concurrently suppressing TGF‐β1‐induced myofibroblast activation. This coordinated intervention normalized the expression of key fibrotic markers, specifically α‐SMA, Col1A1, and FN1, to levels indistinguishable from the control groups.

In a rat IUA model, the CeTA@GPP hydrogel simultaneously regulates the expression of genes related to inflammation, oxidative stress, and fibrosis at the transcriptional level. The hydrogel directly scavenges excess ROS in the pathological uterine microenvironment, thereby alleviating the compensatory activation of the endogenous antioxidant system and effectively restoring the ability of uterine tissue to scavenge O_2_•^−^ and H_2_O_2_. Furthermore, the CeTA@GPP hydrogel promotes macrophage polarization toward the M2 phenotype, effectively inhibits the fibrotic process driven by the TGF‐β1 signaling pathway, including blocking myofibroblast transformation and reducing excessive Col1A1 deposition, while simultaneously improving endometrial receptivity.

The therapeutic potential of CeTA@GPP hydrogel is evidenced by: (1) minimally invasive intrauterine delivery that obviates surgical implantation and removal procedures—the hydrogel's shear‐thinning rheology permits injection through standard clinical catheters while enabling rapid in situ barrier formation; (2) biodegradability of both cerium‐tannic acid complexes and gelatin‐based matrices, eliminating foreign body reactions and chronic inflammation; (3) self‐regulating therapeutic release via ROS‐responsive mechanisms that amplify drug delivery during inflammatory exacerbations while minimizing exposure in healthy tissues and healing phases.

Beyond IUA, this integrated strategy of barrier protection and active multi‐target regulation offers translational potential for related gynecological disorders including endometriosis (oxidative stress‐driven ectopic lesion formation) and pelvic inflammatory disease [76, 77, 78]. The modular platform design accommodates straightforward substitution or combination of alternative nanozymes, or incorporation of complementary therapeutics, enabling personalized interventions tailored to specific pathological contexts.

## 4 Experimental Section

### 4.1 Synthesis and Characterization of CeTA

CeTA was synthesized using a self‐assembly method as described previously with some modifications [47]. Briefly, 250 mg of Ce(NO_3_)_3_·6H_2_O was dissolved in 50 mL sterile double‐distilled water and stirred in a 50°C water bath for 15 min. Concurrently, 500 mg of TA was dissolved in 50 mL sterile double‐distilled water with stirring. Under a 50 °C water bath, the Ce(NO_3_)_3_·6H_2_O solution was slowly added dropwise to the TA solution. NaOH solution (1 M) was then added to adjust the pH to 10, and the reaction was allowed to proceed for 3 h. The synthesized CeTA was collected by centrifugation at 10,000 rpm for 10 min, washed three times with sterile double‐distilled water, and finally freeze‐dried into a powder.

The surface chemical composition and ionic valences were determined by X‐ray photoelectron spectroscopy (XPS, Escalab Xi+, Thermo Fisher Scientific, USA). The coordination interaction between TA and Ce was verified via Fourier transform infrared spectroscopy (FTIR, Nicolet iS50, Thermo Fisher Scientific, USA).

The hydrodynamic diameter of CeTA was measured using a dynamic light scattering instrument (Shenzhou 100v2, China). The surface morphology of CeTA was observed via field emission transmission electron microscopy (FE‐TEM, Talos F200X, Thermo Fisher Scientific, USA), with simultaneous energy dispersive spectrum (EDS) analysis. CeTA was dispersed in deionized water, phosphate‐buffered saline (PBS, 0.01 M, pH 7.4), DMEM/F12 medium, or FBS, and the zeta potential was measured in each solution using a zeta potential analyzer (NanoBrook Omni, USA).

To determine Ce and TA content in CeTA, Ce was quantified using an inductively coupled plasma optical emission spectrometer (ICP‐OES, iCAP 7200, Thermo Fisher Scientific, USA). TA content was measured via the Folin‐Phenol reagent [79]. Specifically, 0.5 mL of TA at a concentration gradient was mixed with 0.5 mL of Folin‐Phenol reagent (0.25 M) and allowed to stand for 3 min. Subsequently, 1 mL of 15% Na_2_CO_3_ was added, and the mixture was allowed to stand for 30 min. After centrifugation at 3500 rpm for 3 min, the absorbance of the supernatant was measured at 760 nm using a microplate reader (FlexStation 3, Molecular Devices, USA). A standard curve was established from the TA concentration series. Finally, CeTA was dispersed in deionized water and analyzed following the above method and the TA content was calculated based on the standard curve.

### 4.2 Enzyme‐Like Activity Assay of CeTA

To assess the antioxidant enzyme‐like activity of CeTA, the Total SOD Activity Assay Kit (S0109, Beyotime, China) and Catalase Assay Kit (S0051, Beyotime, China) were used to measure SOD‐like activity and CAT‐like activity at different CeTA concentrations (10, 50, 100, 200, and 400 µg mL^−1^). Experiments were strictly performed according to the kit instructions. After reacting each CeTA concentration with the corresponding working solution, absorbance was measured at 560 nm (SOD) and 520 nm (CAT) wavelengths using a microplate reader.

### 4.3 Synthesis and Characterization of Gel‐PBA and CeTA@GPP Hydrogel

Gel‐PBA was synthesized using the EDC/NHS method [43]. Subsequently, CeTA at a specific concentration was mixed with 10% PVA in equal proportion and stirred until homogeneous. Finally, mix the mixture with an equal volume of 6% Gel‐PBA solution and stir for a few seconds to crosslink and form the injectable hydrogel CeTA@GPP. The chemical structure of Gel‐PBA was characterized by 1H nuclear magnetic resonance (NMR) spectroscopy (Bruker AVANCE III HD 400, USA) and FTIR. After freeze‐drying the hydrogel for 24 h, field emission scanning electron microscope (FE‐SEM, Zeiss Merlin, Germany) was applied to analyze the microstructure of the hydrogel surface.

Rheological properties of the hydrogel were analyzed using a TA rheometer (TA‐DHR20, USA), including reaction time scanning (0–900 s, constant 5% strain, constant 10 Hz), frequency scanning (0.1–100 rad s^−1^, constant 5% strain), and strain scanning (0.1%–1000% strain, constant 10 Hz). To evaluate the self‐healing properties of Gel‐PBA hydrogel, we performed alternating strain cycle scans: one cycle consisted of a 120 s scan at 1% small strain followed by a 120 s scan at 300% large strain, repeated for five cycles.

To observe the ROS responsiveness and drug release behavior of CeTA@GPP hydrogel, 0.08% alizarin red staining solution or CeTA suspended solution was mixed in equal proportions with 10% PVA, followed by cross‐linking with 6% Gel‐PBA to form hydrogel. Equal volumes of these hydrogels were placed in PBS and 0.03% H_2_O_2_, respectively, to observe their states.

To evaluate the release rate of TA from CeTA@GPP hydrogel, the hydrogels were immersed in PBS or 0.03% hydrogen peroxide solution at 37°C. Release solutions were collected at specific time points (1, 2, 4, 8, 12, 24, 36 h and days 2–8), with an equal volume of fresh solution replenished each time. Release concentrations were determined via microplate reader and calculated based on the TA standard curve. The release rate was calculated using the following formula: Cumulative release rate (%) = (Cumulative released amount / Original amount) × 100%.

### 4.4 In Vitro Cytocompatibility Evaluation

The cytocompatibility of CeTA, GPP, and CeTA@GPP hydrogel were evaluated using a CCK8 cell viability kit (C0038, Beyotime, China) and live/dead assay kit (C2015M, Beyotime, China). In brief, mouse bone marrow‐derived mesenchymal stem cells (mBMSCs) were seeded at 100,000 cells/mL in 96‐well plates. After 24 h of adhesion, cells were treated with different concentrations of CeTA or GPP extract for 1, 3, and 5 days, followed by cytotoxicity assays using CCK‐8 solution or Calcein AM/PI solution (n = 3). For the CCK8 assay, the absorbance of the supernatant at 450 nm was measured using a microplate reader. For live/dead staining experiments, observations were performed using a laser confocal microscope (Zeiss LSM980, Germany). Live cells exhibited green fluorescence, while dead cells displayed red fluorescence.

### 4.5 Effects of CeTA@GPP Hydrogel on ROS and NO Levels and Macrophage Polarization in RAW264.7

The RAW 264.7 cells (Catalog No. CL‐0190) used in this study were provided by Wuhan Procell and are available from ATCC. This cell line (RRID: CVCL_0493) was derived from a male animal and originated from tumors induced by the Abelson murine leukemia virus. Cells were used in the experiments between passages 3–8, ensuring they were in the exponential growth phase. Following the RAW264.7 macrophage M1 polarization induction protocol reported in the published literature [80]. In brief, RAW264.7 cells were seeded at a density of 2 × 10^5^ cells mL^−1^ in 6‐well plates (or in a culture system with a cover slip). When cell density reached 70%, a 24 h co‐induction with 100 ng/mL LPS and 20 ng mL^−1^ IFN‐γ was performed to establish an M1‐polarized RAW264.7 macrophage model. The experiment was designed with five groups (n = 3): a blank control group (blank), an LPS + IFN‐γ co‐induction group (LPS + IFN‐γ), and three intervention groups. After LPS + IFN‐γ induction, these intervention groups were switched to GPP extract (GPP), CeTA suspended solution (CeTA), and CeTA@GPP hydrogel extract (CeTA@GPP), respectively, and cultured for an additional 24 h.

ROS and NO levels in RAW264.7 cells were detected using a ROS assay kit (S0034S, Beyotime Biotech, China) and DAF‐FM DA Probe (S0019S, Beyotime Biotech, China), respectively. Procedures strictly followed the manufacturer's instructions. Briefly, after washing cells in PBS (n = 3), 10 µM DCFH‐DA ROS probe or 5 µM DAF‐FM DA NO probe was added and cultured at 37°C in the dark for 30 min. After washing away residual probes, Hoechst 33342 was added for 10 min to stain cell nuclei. Following thorough washing, cell monolayers were transferred to an inverted confocal dish for observation and imaging using a laser scanning confocal microscope. Images were analyzed using software such as ImageJ to measure the average fluorescence intensity per cell.

To further analyze the effect of CeTA@GPP hydrogel on M1/M2 polarization of RAW264.7 macrophages, the protein expression levels of the M1 marker iNOS and the M2 marker Arg‐1 were detected by WB (n = 3). The experimental results were analyzed using software such as ImageJ.

### 4.6 RAW264.7 Mitochondrial Membrane Potential Detection

The decline in mitochondrial membrane potential is a characteristic event in the early stages of apoptosis. Using the JC‐1 fluorescent probe (C2006, Beyotime Biotech, China) to detect changes in mitochondrial membrane potential. In normal cell mitochondria, JC‐1 exists as aggregates, exhibiting bright red fluorescence. If the mitochondrial membrane potential decreases, JC‐1 exists as monomers, presenting green fluorescence. After treatment of cells in each group (n = 3), mitochondrial membrane potential changes were rigorously assessed following protocol instructions. Experiments were observed and imaged using a laser confocal microscope, with average fluorescence intensities of JC‐1 monomers and aggregates analyzed via software such as ImageJ.

### 4.7 Cellular Uptake of CeTA

We tested RAW264.7 cell uptake of CeTA in vitro. RAW264.7 cells were induced with LPS and IFN‐γ, then cultured with Cy5.5‐labeled CeTA for 24 and 48 h. After PBS washing, cells were stained with Hoechst 33342 for 10 min, and CeTA uptake was observed using a laser confocal microscope.

### 4.8 Anti‐Fibrotic Effects of CeTA@GPP Hydrogel on hESCs In Vitro

HESCs (Catalog No. CP‐H208), which were isolated from human uterine tissue, were provided by Wuhan Procell. Cells were used in the experiments between passages 3–5, ensuring they were in the exponential growth phase. Following a previously validated protocol for inducing fibrosis in hESCs^72^, hESCs were seeded at a density of 2 × 10^5^ cells per well in 6‐well plates and treated with 10 ng mL^−1^ TGF‐β1 for 48 h to induce cellular fibrosis. Following the removal of the induction medium, we added culture media containing GPP extract (GPP), CeTA suspended solution (CeTA), or CeTA@GPP hydrogel (CeTA@GPP) respectively, and continued culturing for 24 h. The normal group(blank) and TGF‐β1‐induced group(TGF‐β1) were included as reference groups.

Real‐Time PCR(RT‐PCR) was applied to detect mRNA expression levels of α‐SMA, FN1, and Col1A1. Subsequently, IF staining was performed to observe the localization and expression of α‐SMA, FN1, and Col1A1. Concurrently, FN1 and Col1A1 proteins were quantitatively analyzed through WB analysis.

### 4.9 Scratch Assay

HESCs were seeded in six‐well plates and treated with TGF‐β1 for 48 h. A scratch wound was produced on the cell surface using a pipette tip, after which the hESCs were incubated in the conditioned medium(n = 3). The scratch area was photographed using microscopy at different time points (0, 12, 24, 36 h), and the scratch healing rate was calculated by comparing the closed area with the initial wound area.

### 4.10 Treatment of IUA With CeTA@GPP Hydrogel

All animal experimental procedures were approved by the Animal Ethics and Welfare Committee of Guangzhou Shuiyuntian (approval no. SYT2025052) and conducted in strict accordance with the Animal Research: Reporting of In Vivo Experiments (ARRIVE) guidelines. During the acclimation phase, all experimental rat cages were maintained under a 12 h light/dark cycle.

Female Sprague‐Dawley rats (180–220 g, 6–8 weeks old) were selected for this experiment. The animals were housed in a pathogen‐free environment with free access to food and water. The IUA rat models were constructed by using a combined mechanical injury and infection method, as previously described [81]. All experiments were conducted under aseptic conditions. Briefly, a total of 20 female Sprague‐Dawley rats with regular estrous cycles were randomly divided into four groups (5 rats per group): sham surgery group (Sham), IUA model group (IUA), IUA + GPP group (GPP), and IUA + CeTA@GPP group (CeTA@GPP). Rats were anesthetized with 2% isoflurane and maintained on a 37°C heating pad to preserve normothermia. After the deep anesthesia, the lower abdomen was sterilized and incised with a 3 cm longitudinal incision to expose both sides of the uterus. A 2‐mm incision was made near the cervix on both sides of the uterus. A uterine curette was inserted through the incision into the uterine cavity and scraped until the endometrium became noticeably congested and roughened. Subsequently, 1–0 surgical suture pre‐soaked in a 6 mg L^−1^ LPS solution at 4°C for 24 h was implanted into the uterine cavity. The suture tail was led through the abdominal wall and secured to the skin surface for removal 48 h post‐surgery. After placing the suture containing LPS, 100 µL of GPP or CeTA@GPP hydrogel was injected into the uterine cavity through a syringe along the uterine incision, while the IUA group did not receive any injection [82]. Subsequently, the uterus and abdomen were sutured layer by layer. After the operation, rats were placed in a warm cage to maintain their body temperature until they regained consciousness. All rats received continuous penicillin (50,000 units per day) injections for 3 days after the operation. The rats were sacrificed at 2 weeks after the operation and uterine samples were obtained for subsequent experimental analysis.

To observe the in vivo degradation of the CeTA@GPP hydrogel, Cy5.5‐labeled CeTA was mixed with GPP and injected into the uterine cavity of rats. The degradation of this hydrogel system was evaluated at days 1, 3, 6, and 14 using in vivo imaging (Tanon ABL‐X3, China).

### 4.11 Evaluation of the Effect of Endometrial Repair

After the operation, gross observation of the uterus was performed based on the following aspects: uterine shape, congestion degree, surface roughness, and tissue swelling. For histological evaluation, uterine tissues were fixed, embedded, and sectioned. Hematoxylin/Eosin (H&E) and Masson's trichrome staining were adopted to evaluate endometrial thickness, the number of glands, and the percentage of fibrosis areas using imageJ software. Additionally, IF staining was used to detect α‐SMA, Col1A1, Ki67, and VEGFA. IF images were randomly selected from three fields of view for fluorescence intensity analysis using ImageJ. Immunohistochemical staining was conducted to evaluate the expression of markers related to endometrial regeneration, including estrogen receptor (ER, BF8047, Affinity) and progesterone receptor (PR, AF6106, Affinity). All the stained sections were imaged using a digital pathology scanning system (Panoramic 250, 3D Histech, Hungary) and analyzed with ImageJ software.

### 4.12 Evaluation of Uterus Fertility

Twelve female Sprague‐Dawley rats (180–220 g, 6–8 weeks old) were randomly divided into four groups (n = 3) and treated with IUA modeling and therapy according to the aforementioned methods. After 28 days post‐surgery, all female rats were naturally mated with healthy male rats at a 2:1 ratio. The rats were euthanized at mid‐gestation, and uteri were collected to analyze the number of embryos.

### 4.13 Statistical Analysis

Data visualization and statistical analyses were performed using GraphPad Prism 10.1.2 software (USA). The normality of data in each group was assessed using the Shapiro‐Wilk test, and all datasets satisfied the assumption of normal distribution. Statistical significance was evaluated using a paired t‐test or one‐way analysis of variance (ANOVA). For data with unequal variances, Welch's ANOVA and Brown‐Forsythe ANOVA were applied. When an overall statistically significant difference was detected (*p < *0.05), Tukey's HSD test was further performed for multiple‐comparison correction. Experimental data are presented as mean ± standard deviation (SD), and all data were obtained from three or more independent samples. The symbols *, **, ***, and **** in the figures indicate significant differences between groups. *p < 0.05, **p < 0.01, ***p < 0.001, ****p < 0.0001.

## Author Contributions

P.C. conceptualized the study, developed the methodology, analyzed the data, wrote the original draft, and validated the experimental results. J.S. assisted with the animal experiments and data verification, and reviewed and edited the writing. Y.L. contributed to the cell experiments and animal experiments. M.C. contributed to scientific discussions of this work. Z.L. assisted with the characterization of the hydrogel. B.Q. contributed to the animal experiments. Y.F. acquired funding, provided resources, supervised the work, and reviewed and edited the writing. X.S. provided resources, supervised the work, and reviewed and edited the writing. X.L. conceptualized the study, acquired funding, provided resources, developed the methodology, performed visualization, supervised the work, and reviewed and edited the writing. All authors contributed to the manuscript and approved the final version. The authors used Deepseek V4 to assist with language editing and manuscript polishing. The authors take full responsibility for the content of this work.

## Funding

This work was financially supported by the National Natural Science Foundation of China (32301136, 82571911), the National Key Research and Development Program of China (2024YFC2706604, 2024YFA1106902), the Guangdong Major Project of Basic Research (2025B0303000014), and the Fundamental Research Funds for the Central Universities.

## Conflicts of Interest

The authors declare no conflicts of interest.

## Supporting information




**Supporting File 1**: advs77510‐sup‐0001‐SuppMat.docx.

**Supporting File 2**: advs77510‐sup‐0002‐SuppMat.docx.

## Data Availability

The data that support the findings of this study are present in the manuscript and supporting information. The raw RNA‐seq data generated in this study have been deposited in the Gene Expression Omnibus (GEO) database under accession number GSE339272. Other data are available from the corresponding author upon reasonable request.
