# Supplementary material for: An Injectable ROS‐Responsive Nanozyme Hydrogel Regulates the Uterine Microenvironment to Prevent Intrauterine Adhesions
Source: Adv Sci (Weinh). 2026 Aug 30:e77510. Online ahead of print. doi: 10.1002/advs.77510 (PMC13526704; doi:10.1002/advs.77510)
Supplement: Supplementary file 1 [file ADVS-9999-e77510-s001.docx]

Supporting Information

**An Injectable ROS-Responsive Nanozyme Hydrogel Regulates the Uterine Microenvironment to Prevent Intrauterine Adhesions**

*Peixian Cheng^1^, Jian Song^3^, Yuxin Li^1^, Ming Chen^4^, Zeshen Liang^5^, Binyao Qiu^6^, Xuemin Liu^2*^, Xuetao Shi^5*^, Yong Fan^1*^*

**Supplementary Methods**

1 Synthesis of Gel-PBA

Gel-PBA was synthesized using the EDC/NHS method. Briefly, 10 g of gelatin was dissolved in 200 mL of MES buffer (0.05 M, pH 5.5) at 37°C in a water bath. Simultaneously, dissolve 9.6 g EDC.HCl, 2.3 g NHS, and 4 g 3-COOH-PBA in 800 ml MES buffer. Stir at 37°C for 30 minutes to activate. Once gelatin is fully dissolved, slowly add this solution into the above mixture. Continue stirring at 37°C for 48 h. Subsequently, dialyze the reaction solution in deionized water using a dialysis bag with a molecular weight cutoff of 8000–14000 Da for 3 days. Freeze-dry the dialyzed solution to obtain white, sponge-like Gel-PBA.

2 Real-Time qPCR (RT-qPCR)

Total RNA was purified from the cultured cells. The RNA was subsequently reverse transcribed into cDNA via a reverse transcription kit. The reverse transcription process included incubation at 25°C for 5 minutes, 42°C for 30 minutes, and 85°C for 5 seconds. Quantitative PCR (qPCR) amplification was performed via SYBR Green qPCR Master Mix and gene-specific primers. The amplification process consisted of predenaturation, denaturation, and annealing/extension steps, with fluorescence signals collected at the end of each cycle. Gene expression changes were analyzed via the 2^(-ΔΔCt) method, with GAPDH or β-actin used as the internal reference. Each RNA sample was analyzed in triplicate to ensure reproducibility. The sequences of primers used for qPCR are provided in Supplementary Table S1.

3 Western Blot (WB)

The cells were washed twice with ice-cold phosphate-buffered saline (PBS) and lysed on ice using RIPA buffer (Beyotime) containing protease and phosphatase inhibitors (Roche). Lysates were centrifuged at 12,000 × g for 15 minutes at 4°C. The supernatant was collected, and total protein concentration was determined using a BCA Protein Assay Kit (Beyotime). Equal amounts of protein (20 μg per sample) were denatured in SDS loading buffer, separated by SDS-polyacrylamide gel electrophoresis (SDS-PAGE) on 10-12.5% gels, and subsequently transferred onto polyvinylidene difluoride (PVDF) membranes (Millipore). Membranes were blocked with 5% non-fat dry milk in Tris-buffered saline containing 0.1% Tween-20 (TBST) for 2 hours at room temperature. After blocking, membranes were incubated with primary antibodies diluted in blocking solution either for 4 hours at room temperature or overnight at 4°C with gentle agitation. Following extensive washing with TBST, membranes were incubated with appropriate horseradish peroxidase (HRP)-conjugated secondary antibodies diluted in blocking solution for 1.5 hours at room temperature or for 1 hour at 30°C. Protein bands were visualized using an enhanced chemiluminescence (ECL) detection kit (Beyotime) and imaged with a chemiluminescence imaging system. Band intensities were quantified using ImageJ software. Protein expression levels of target proteins were normalized to the level of β-actin.

4 Immunofluorescence Staining

The cells were fixed with 4% paraformaldehyde at room temperature for 20 minutes and were washed with PBS. Permeabilization was performed with 0.2% Triton X-100 at room temperature for 20 minutes, followed by PBS washing. Subsequently, cells were blocked with 3% BSA at room temperature for 60 minutes, then were incubated with the primary antibody overnight at 4℃. After washing, the secondary antibody was added and incubated at room temperature in the dark for 1 hour. Following PBS washing, DAPI stain was added for 10 minutes. Anti-fluorescence quencher was added dropwise to the slide, then the cell mount was removed and inverted onto a new slide.

For endometrial tissue samples, the endometrium was fixed in paraformaldehyde, dehydrated in a graded ethanol series, and embedded in paraffin. The paraffin-embedded tissues were sectioned into 5 µm slices, followed by deparaffinization and antigen retrieval. Endogenous peroxidase activity was blocked with 3% hydrogen peroxide solution. The sections were permeabilized in 0.5% Triton X-100 at 25℃ for 20 minutes and washed three times with PBS. Blocking was performed at room temperature with 3% BSA for 1 hour. Subsequently, primary antibodies were added to the sections, and the samples were incubated overnight at 4℃. After three washes, the samples were incubated with secondary antibodies diluted in PBS at room temperature for 1 hour. The TSA reagent kit was used to amplify the fluorescent signal, and then the experimental operation from BSA blocking to staining with the second antibody was repeated. The sections were then washed three times and counterstained with DAPI for 10 minutes to visualize the nuclei. Finally, an appropriate amount of anti-fluorescence quencher was added and the slide was covered.

Observation and imaging were performed using a laser confocal microscope, and fluorescence intensity was analyzed using software such as ImageJ.

The primary antibodies used included anti-alpha smooth muscle actin (1:800, ab18147, Abcam), anti-Fibronectin (1:200, 15613-1-AP, Proteintech), anti-Col1A1 (1:800, GB11022-3-100, Servicebio), anti-Nrf2 (1:100, AF0639, Affinity), anti-iNOS (1:100, AF0199, Affinity), anti-CD206 (1:4000, GB155273, Servicebio), anti-VEGFA (1:200, ab1316, Abcam) and anti-Ki67 (1:200, AF0198, Affinity) antibodies.

The secondary antibodies used included goat anti-rabbit Alexa Fluor 488 (1:400, ab150077, Abcam), goat anti-rabbit Alexa Fluor 555 (1:400, ab150078, Abcam), Alexa Fluor 488-conjugated goat anti-mouse IgG (1:400, GB25301, Servicebio), Goat anti-mouse IgG H&L (HRP) (1:800, ab205719, Abcam) and Goat anti-rabbit IgG H&L (HRP) (1:800, ab205718, Abcam).

5 H&E and Masson Staining

The tissue samples were fixed in 4% paraformaldehyde for 24 hours and then sectioned. After fixation, the samples were dehydrated, embedded in paraffin, and cut into 5 μm-thick cross-sections. To evaluate pathological alterations in the endometrium, H&E staining and Masson staining were performed. The sections were deparaffinized, rehydrated, rinsed with deionized water, and stained with H&E and Masson staining solutions (Servicebio, China). Masson staining was specifically used to assess fibrosis levels. Images of the stained sections were captured via a digital slide scanner (Panoramic 250, 3D Histech, Hungary), and the results were analyzed quantitatively via ImageJ software.

6 Immunohistochemical Staining

After fixation, the paraffin-embedded tissue sections were subjected to deparaffinization, permeabilization, and antigen retrieval. The sections were sequentially immersed in an environmentally friendly deparaffinizing solution, absolute ethanol, and distilled water to complete the deparaffinization and rehydration process. Antigen retrieval was performed using EDTA antigen retrieval solution. Endogenous peroxidase activity was blocked with 3% hydrogen peroxide solution, and nonspecific binding was blocked by incubating the sections in 3% BSA for 1 hour. The sections were then incubated with the primary antibody mixture overnight at 4 ℃, followed by three washes and the incubation with secondary antibodies for 50 minutes. DAB was used for chromogenic detection, and the sections were counterstained with hematoxylin for 3 minutes. Finally, the sections were dehydrated, cleared through a graded alcohol and xylene series, and mounted with neutral mounting medium. The stained sections were observed under a bright-field microscope to interpret the results. Images of the immunohistochemically stained sections were acquired using a digital pathological slide scanner (Panoramic 250, 3D Histech, Hungary) and analyzed using ImageJ software for data processing and quantification.

7 RNA Sequencing and Bioinformatics Analysis

Total RNA was extracted from rat uterine tissue samples. RNA concentration and purity were subsequently assessed, and RNA integrity was evaluated using an Agilent 2100 Bioanalyzer. For mRNA library construction, poly(A)-tailed mRNA was first enriched from total RNA using oligo(dT) magnetic beads. The enriched mRNA was then fragmented into approximately 300 bp fragments by ion-mediated fragmentation. cDNA was synthesized using the fragmented RNA as a template. After PCR amplification, library fragments with a final library size of approximately 450 bp were selected. Library quality was then assessed using an Agilent 2100 Bioanalyzer. Subsequently, libraries with different index sequences were pooled in appropriate proportions according to their effective concentrations and the required sequencing depth. After RNA extraction, purification, and library construction, paired-end sequencing was performed on an Illumina sequencing platform using next-generation sequencing technology.

The raw sequencing data were first subjected to quality control and filtering. The filtering criteria mainly included removal of reads containing 3′ adaptor sequences using fastp, removal of reads with an average quality score lower than Q20, removal of reads containing more than 10% ambiguous bases, and removal of reads shorter than 20 bp after adaptor trimming and quality filtering. High-quality clean reads were obtained after filtering. FastQC was then used to further evaluate base quality. Clean reads were aligned to the rat reference genome Rattus_norvegicus.mRatBN7.2.dna.toplevel.fa using HISAT2. The genomic distribution of mapped reads was summarized, and the number of mapped reads and transcript length were normalized. HTSeq was used to count the read counts mapped to each gene, which were used as the raw gene expression values. To correct for the effects of sequencing depth and gene length on expression estimation, gene expression levels were normalized using FPKM (Fragments Per Kilobase of transcript per Million mapped fragments).

To evaluate sample consistency and the reliability of biological replicates, Pearson correlation coefficients were calculated between all pairs of samples based on FPKM values. Principal component analysis (PCA) was also performed to assess sample clustering and potential batch effects.

Differential expression analysis was performed using DESeq2. Genes with |log2FoldChange| ≥ 1 and P value < 0.05 were considered differentially expressed genes. Subsequently, functional annotation was performed for the differentially expressed genes between comparison groups, followed by GO and KEGG pathway enrichment analyses. Enriched functions or pathways with P value < 0.05 were considered statistically significant.

**Supplementary Tables**

Table S1. Information on the primers used for the targeted genes. (Samples: HESCs)

| **Gene** | **Forward** | **Reverse** |
| --- | --- | --- |
| GAPDH | CCTCGTCCCGTAGACAAAATG | TGAGGTCAATGAAGGGGTCGT |
| β-actin | GTGACGTTGACATCCGTAAAGA | GTAACAGTCCGCCTAGAAGCAC |
| α-SMA | GTACCACCATGTACCCAGGC | GAAGGTAGACAGCGAAGCCA |
| FN1 | AATCAGCAGTGGGAACGGAC | CACTTGTCGCCAATCTTGTAGG |
| Col1A1 | CTCCCAGAACATCACCTATCACT | GGAGGTCTTGGTGGTTTTGTATT |

Table S2. Acronym Table

| **Acronym** | **Full Term** |
| --- | --- |
| IUA | Intrauterine Adhesions |
| CeTA | Cerium-Tannic Acid |
| Gel-PBA | phenylboronic Acid-Modified Gelatin |
| PVA | Polyvinyl Alcohol |
| PBS | Phosphate-Buffered Saline |
| FBS | Fetal Bovine Serum |
| DMEM | Dulbecco's Modified Eagle Medium |
| LPS | Lipopolysaccharide |
| IFN-γ | Interferon - Gamma |
| INOS | Inducible Nitric Oxide Synthase |
| Arg-1 | Arginase 1 |
| TGF-β1 | Transforming Growth Factor Beta 1 |
| α-SMA | Alpha - Smooth Muscle Actin |
| FN1 | Fibronectin 1 |
| Col1A1 | Collagen Type I Alpha 1 Chain |
| VEGFA | Vascular Endothelial Growth Factor A |

**Supplementary Figuers**

Figure S1. (A) FE-TEM image of CeTA. Scale bar = 200 nm. (B-D) The hydrodynamic diameter distribution of CeTA in FBS, DMEM/F12, and H_2_O. (E) The hydrodynamic diameter changes of CeTA in DMEM/F12 basal medium over 0, 1, 3, and 5 days.

Figure S2. In vitro biocompatibility evaluation of CeTA. (A-C) CCK-8 assay showing mBMSCs proliferation levels at 1, 3, and 5 days (n = 5). All data are expressed as mean ± SD. (D) Live/dead fluorescence staining images (live cells are green, dead cells are red) (n = 3). Scale bar: 100 µm.

Figure S3. Rheological tests of the GPP hydrogel and CeTA@GPP hydrogel. (A) Reaction time scanning (0-900 s, constant 5% strain, constant 10 Hz). (B) Oscillatory frequency sweep (0.1-100 rad/s, constant 5% strain).

Figure S4. (A) In vitro degradation curves of CeTA@GPP hydrogel immersed in PBS and H_2_O_2_, respectively. (B) FE-SEM images of CeTA@GPP hydrogel immersed in PBS and H_2_O_2_ on days 1, 3, and 5, respectively. Scale bar: 100 µm. (C) FE-SEM images and EDS elemental mapping images of CeTA@GPP hydrogel immersed in PBS on days 0 and 5. Scale bar: 20 µm.

Figure S5. In vitro biocompatibility evaluation of GPP hydrogel and CeTA@GPP hydrogel. (A, C) CCK-8 assay showing mBMSCs proliferation levels at 1, 3, and 5 days (n = 5). (B, D) Live/dead fluorescent staining images of mBMSCs after treatment with GPP or CeTA@GPP (live cells are green, dead cells are red) (n = 3). Scale bar: 100 µm. All data are expressed as mean ± SD.

Figure S6. Cellular Uptake of CeTA. Immunofluorescence microscopy images showed Cy5.5-CeTA expression (red) in macrophages. Nuclei were stained with Hoechst 33342 (blue). Scale bar: 20 µm.

Figure S7. Effects of CeTA@GPP hydrogel on the migration of hESCs. (A-D) Representative bright-field images and quantitative analysis of hESCs wound healing after treated with GPP hydrogel, CeTA and CeTA@GPP hydrogel at 0, 12, 24 and 36 h (n = 3). Scale bar = 1000 µm. All data are expressed as mean ± SD (**P* < 0.05, ***P* < 0.01, ****P* < 0.001, *****P* < 0.0001).

Figure S8. Therapeutic evaluation of the use of CeTA@GPP hydrogel for the treatment of IUA. (A) In vivo fluorescence imaging showing the distribution of Cy5.5-labeled CeTA@GPP hydrogel at 1, 3, 6, and 14 days after surgery. (B) The gross observation of uterus after 14 days of surgery. Scale bar: 1 cm. (C) Representative images of Masson trichrome staining of rat uteri sections at 14 days postoperatively (n = 3). (D-F) Endometrial thicknesses, glands number and fibrotic area were calculated in each group (n = 3). All data are expressed as mean ± SD (**P* < 0.05, ***P* < 0.01).

Figure S9. (A-C) SOD and CAT activities in uterine tissue homogenates of rats from each group (n = 3). All data are expressed as mean ± SD (**P* < 0.05, ***P* < 0.01, ****P* < 0.001).

Figure S10. (A-C) Volcano plot of DEGs in GPP vs. IUA, CeTA@GPP vs. GPP and CeTA@GPP vs. Sham (purple: upregulated; green: downregulated; gray: nonsignificant). (D) KEGG pathway enrichment of CeTA@GPP vs. GPP. Bubble size: gene count. Color: Rich factor. (Left side of the x-axis: downregulated DEGs enrichment pathways; Right side: Upregulated DEGs enrichment pathways). (E) GO biological process (BP), cellular component (CC) and molecular function (MF) enrichment for the CeTA@GPP vs. IUA. Bubble size: gene count.

Figure S11. Differential expression of inflammation related genes (A), oxidative stress related genes (B), and fibrosis related genes (C) among four groups of uterine tissues.

Figure S12. The result of PPI analysis between the CeTA@GPP group and the IUA group.

Figure S13. Biosafety analysis of major organs after different treatments. HE staining revealed histological changes in the heart, liver, spleen, lung and kidney from the Sham, IUA, GPP and CeTA@GPP groups. No significant pathological damage was observed in the major organs across the groups, indicating good biosafety under different treatment conditions. Small, round cells with dense nuclear staining and minimal cytoplasm are characteristic of inflammatory cells. Scale bar = 200 μm.

Figure S14. (A–C) Serum levels of ALT, AST, and CREA in rats from the different groups (n = 5). All values remained within the normal reference ranges.

Figure S15. Comparison of body weights of rats in each group (n = 3). No statistically significant differences were observed among the groups.
